# Studies Relevant to the Functional Model of Mo-Cu CODH: In Situ Reactions of Cu(I)-L Complexes with Mo(VI) and Synthesis of Stable Structurally Characterized Heterotetranuclear Mo^VI^_2_Cu^I^_2_ Complex

**DOI:** 10.3390/molecules28083644

**Published:** 2023-04-21

**Authors:** Umesh I. Kaluarachchige Don, Ahmad S. Almaat, Cassandra L. Ward, Stanislav Groysman

**Affiliations:** 1 Department of Chemistry, Wayne State University, 5101 Cass Avenue, Detroit, MI 48202, USA; gp2280@wayne.edu (U.I.K.D.);; 2 Lumigen Instrument Center, Wayne State University, 5101 Cass Avenue, Detroit, MI 48202, USA

**Keywords:** molybdenum, copper, CO dehydrogenase, catechol, biomimetic

## Abstract

In this study, we report the synthesis, characterization, and reactions of Cu(I) complexes of the general form Cu(L)(LigH_2_) (LigH_2_ = xanthene-based heterodinucleating ligand (E)-3-(((5-(bis(pyridin-2-ylmethyl)amino)-2,7-di-tert-butyl-9,9-dimethyl-9H-xanthen-4-yl)imino)methyl)benzene-1,2-diol); L = PMe_3_, PPh_3_, CN(2,6-Me_2_C_6_H_3_)). New complexes [Cu(PMe_3_)(LigH_2_)] and [CuCN(2,6-Me_2_C_6_H_3_)(LigH_2_)] were synthesized by treating [Cu(LigH_2_)](PF_6_) with trimethylphosphine and 2,6-dimethylphenyl isocyanide, respectively. These complexes were characterized by multinuclear NMR spectroscopy, IR spectroscopy, high-resolution mass spectrometry (HRMS), and X-ray crystallography. In contrast, attempted reactions of [Cu(LigH_2_)](PF_6_) with cyanide or styrene failed to produce isolable crystalline products. Next, the reactivity of these and previously synthesized Cu(I) phosphine and isocyanide complexes with molybdate was interrogated. IR (for isocyanide) and ^31^P NMR (for PPh_3_/PMe_3_) spectroscopy demonstrates the lack of oxidation reactivity. We also describe herein the first example of a structurally characterized multinuclear complex combining both Mo(VI) and Cu(I) metal ions within the same system. The heterobimetallic tetranuclear complex [Cu_2_Mo_2_O_4_(μ_2_-O)(Lig)_2_]·HOSiPh_3_ was obtained by the reaction of the silylated Mo(VI) precursor (Et_4_N)(MoO_3_(OSiPh_3_)) with LigH_2_, followed by the addition of [Cu(NCMe)_4_](PF_6_). This complex was characterized by NMR spectroscopy, high-resolution mass spectrometry, and X-ray crystallography.

## 1. Introduction

Molybdenum-copper carbon monoxide dehydrogenase (Mo-Cu CODH) is an air-stable enzyme that catalyzes the oxidation of CO to CO_2_. Mo-Cu CODH is a key enzyme in the metabolism of aerobic carboxidotrophic bacteria (such as *Oligotropha carboxidovorans*), allowing it to use CO as a sole source of energy and carbon [1]. The oxidation of CO is very efficient, and the enzyme detoxifies a significant fraction of total CO from the atmosphere [2]. Thus, there is a longstanding interest in the design of structural and/or functional models which could (a) provide insight into the reaction mechanism, and (b) lead to efficient CO oxidation and potentially CO_2_ reduction catalysts.

**Scheme 1:**
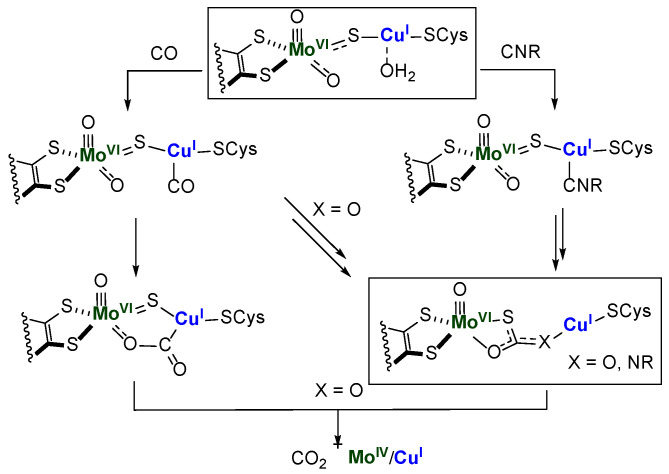
Resting site (oxidized) structure of Mo-Cu CODH, and its proposed reactivity with CO and CNR. (R = tert-butyl, [SC(R) = C(R)S] ligand = pyranopterindithiolene). Structures inside frames have been characterized by X-ray crystallography (X = NR).

The oxidized active site of Mo-Cu CODH contains square-pyramidal Mo(VI) coordinated to pyranopterindithiolene, [3,4] apical and basal oxo groups, and a basal sulfido bridging to a low-coordinate Cu(I) atom, whose coordination is accomplished by cysteinate and weakly bound water at the resting state (Scheme 1) [5,6]. The reduced active site likely contains Mo(IV)-basal hydroxide in an otherwise similar disposition [7]. Spectroscopic studies suggest that the coordination of CO takes place at the Cu(I) center, replacing weakly bound water [8,9]. Computational studies generally agree with the initial coordination of CO to the coordinatively unsaturated Cu(I) site. However, the subsequent routes differ between different mechanisms, suggesting bridging thiocarbonate or carbon dioxide intermediates [10,11,12,13,14,15,16,17]. It is noted that the bridging thiocarbonate hypothesis relies to a large extent on a high-resolution structure of the product of the reaction of Mo-Cu CODH with alkyl isocyanide (CO analog/inhibitor), which undergoes oxidation and insertion into the Cu-S bond to form bridging thiocarbamate [5]. Since the report of the high-resolution structure of the Mo-Cu CODH enzyme, several structural models of Mo-Cu CODH have been reported; in all these models the metals were linked only by bridging sulfido (or oxo and sulfido) ligands [18,19,20,21,22,23,24]. Although selected closely related structural models were able to rationalize structural, electronic, and spectroscopic features of the active site, only a few have explored the reactivity with CO or CO-like substrates [24]. In our previous work, we explored a different approach toward a more robust functional model of Mo-Cu CODH, which brings two metals together using xanthene-based heterodinucleating ligands [25]. Recently, we have shown that the heterodinucleating ligand LigH_2_, which combines catecholate chelate for Mo(VI) and dipicolylamine for Cu(I), allows for the formation of mononuclear Cu(I) and Mo(VI) complexes at the designated sites [26]. The combination of LigH_2_ with both metals forms metastable Mo(VI)-Cu(I) complex (Et_4_N)[CuMoO_3_(Lig)] directly observable in solution by ^1^H NMR and high-resolution mass spectrometry (HRMS). However, our attempts to isolate this complex in a solid state and/or study its reactivity were not successful, as it underwent facile decomposition. In the present work, we explore a different approach toward a functional model, which involves the initial formation of well-defined Cu(I) complexes containing an oxidizable CO-like (or related) ligand (CNR, PR_3_, CN^−^, olefin), followed by treatment with (Et_4_N)_2_[MoO_4_]. We have also explored a different approach towards stable Mo(VI)-Cu(I) complex that involves the use of a different Mo(VI) precursor (Et_4_N)[MoO_3_(OSiPh_3_)]. Herein we report the first structurally characterized Mo(VI)-Cu(I) complex in our system. 

## 2. Results and Discussion

### 2.1. Synthesis and Characterization of Cu(I)-LigH_2_ Complexes Featuring an Additional L Ligand

**Scheme 2:**
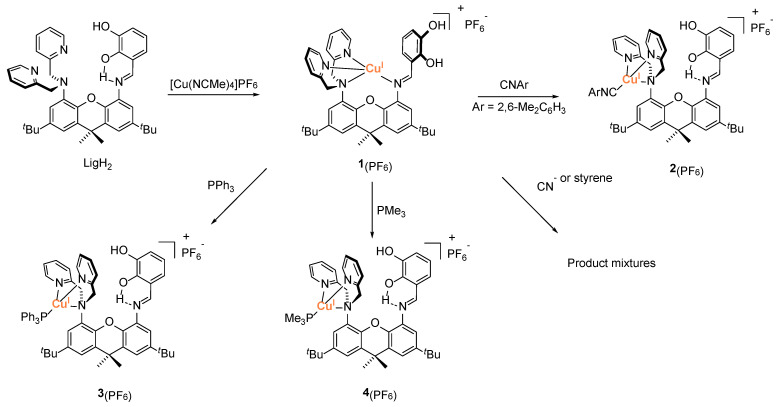
Synthesis of Cu(I) precursors described in this paper.

As described above, we have targeted complexes of the general form [Cu(L)(LigH_2_)](PF_6_), in which L is either directly related to the reactivity of Mo-Cu CODH (L = CNR), or can undergo similar oxidation reactivity (CN^−^, PR_3_, olefin). To access these complexes, we have used previously reported Cu(I) complex [Cu(L)(LigH_2_)](PF_6_) (**1**(PF_6_)) (Scheme 2) as a precursor [24]. Complex **1**(PF_6_) demonstrates strong coordination of Cu(I) by two pyridine and the imine donors; relatively weak interaction with the central amine was observed. A reaction of complex **1**(PF_6_) with a monodentate ligand L is likely to release Cu(I) from the coordination to the imine, while restoring the H-bonding between the catechol proton and the imine [26,27]. 

**Figure 1:**
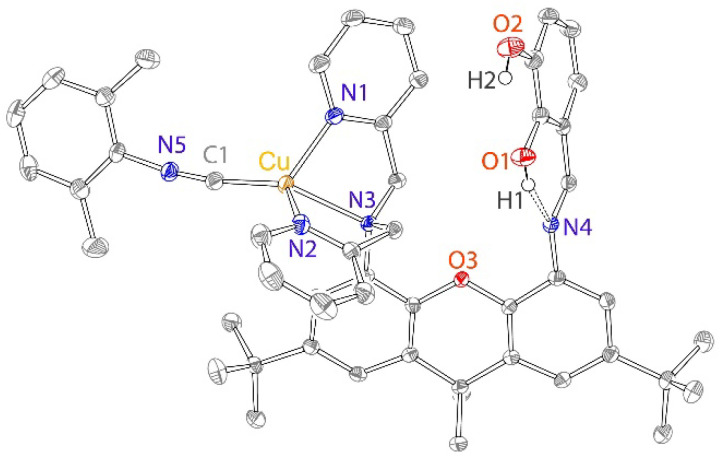
The structure of **2**(PF_6_) with 50% probability ellipsoids. PF_6_ counterion, hydrogen atoms, and co-crystallized solvent (ether) were omitted for clarity.

Treatment of complex **1**(PF_6_) with 2,6-dimethylphenyl isocyanide CN(2,6-Me_2_C_6_H_3_) produced [Cu(CN(2,6-Me_2_C_6_H_3_))(LigH_2_)](PF_6_) (**2**(PF_6_)) in 90% yield as yellow-orange crystals. **2**(PF_6_) was characterized by ^1^H and ^13^C NMR spectroscopy, FT-IR spectroscopy, HRMS, and X-ray crystallography. The coordination of CN(2,6-Me_2_C_6_H_3_) to the metal is supported by the presence of the isocyanide methyl groups at 2.32 ppm in ^1^H NMR spectrum. Notably, methylene protons (NC*H*_2_py) appear as a sharp singlet, suggesting weak coordination of the central amine donor. The IR spectroscopy demonstrates the C≡NAr stretch at 2137 cm^−1^. This value suggests the coordination of an isocyanide to Cu(I) through mostly σ-donation [25,28,29]. HRMS demonstrates a peak at 848.3577 in the positive ion mode (expected *m/z* for **2**^+^ is 848.3595), which agrees well with the expected isotopic distribution (see Supplementary Materials). The X-ray structure of **2**(PF_6_) (Figure 1) confirms the overall composition and is consistent with the spectroscopic features. Coordination of the isocyanide to Cu(I) leads to the release of the metal and the rotation of the Cu(I)-dipicolylamine fragment away from the imine. Copper coordination is replaced by a strong hydrogen bond with one of the catechol-OH groups (OH-N(imine) 1.88 Å). The bond distance between Cu(I) and the central amine is relatively long (Cu1 N1 2.281(1) Å), while the bonds with the pyridine nitrogen are relatively short (2.0289(14) and 2.0274(14) Å). A short bond (1.844(2) Å) is observed between Cu(I) and isocyanide as well. 

We have also targeted copper-phosphine complexes (PR_3_ = PPh_3_ and PMe_3_). The synthesis and structure of [Cu(PPh_3_)(LigH_2_)](PF_6_) (**3**(PF_6_)) habe been reported previously (Scheme 2) [26]. [Cu(PMe_3_)(LigH_2_)](PF_6_) (**4**(PF_6_)), featuring more reactive trimethylphosphine, was obtained by the treatment of **1**(PF_6_) with one equivalent of PMe_3_. The complex was characterized by ^1^H, ^13^C, and ^31^P NMR and HRMS. Our attempts to obtain its solid-state structure were not successful; however, the spectroscopic data for **4**(PF_6_) is consistent with the spectroscopic data for **3**(PF_6_), suggesting a similar structure. Both compounds give rise to a catechol proton at a highly downfield chemical shift, 14.07 ppm for **3**(PF_6_) and 13.93 ppm for **4**(PF_6_), suggesting strong (catechol)OH-N(imine) hydrogen bonding. In both cases, methylene arms protons NC*H*_2_py appear as broad peaks, suggesting weak but not negligible coordination of the central amine. ^31^P NMR spectroscopy demonstrates ^31^*P*Me_3_ signal at −49.0 ppm in **4**(PF_6_). HRMS contains a molecular ion peak for **4**^+^ at *m/z* 793.3295 (expected *m/z* is 793.3302).

In addition to the synthesis of PR_3_ and isocyanide complexes, we have also attempted the synthesis of the mononuclear cyanide complex [Cu(CN)(LigH_2_)] and a mononuclear olefin (styrene CH_2_CHPh) complex. It was previously reported that the reaction of [CuCN]_n_ with LigH_2_ formed a [Cu_2_(CN)_2_(LigH_2_)] complex featuring two coppers and two cyanides: one terminal and one bridging {Cu(μ_2_-CN)CuCN} [26]. To prevent the formation of the [CuCN]_n_ chains (likely resulting from the [CuCN]_n_ precursor), complex **1**(PF_6_) was treated with the combination of NaCN and crown ether (18-crown-6). However, the reaction led to a relatively uninformative NMR spectrum containing broad peaks, which suggested product mixture or dynamic processes which could involve species of varying nuclearities. Our attempts to obtain a pure crystalline product from this reaction were not successful. Similarly, the reaction of **1**(PF_6_) with styrene produced a broad NMR spectrum containing excess styrene. Our attempts to purify this compound by crystallization were not successful as well.

### 2.2. Reactions of Cu-L Complexes (L = CNR, PR_3_, Styrene) with [MoO_4_]^2−^

The reactions of complexes **2**(PF_6_), **3**(PF_6_), and **4**(PF_6_) with (Et_4_N)_2_[MoO_4_] were investigated by adding the equimolar amounts of the acetonitrile solution of molybdate into the stirring acetonitrile solution of the corresponding complex. These reactions were performed similarly to the previously described synthesis of metastable (Et_4_N)[CuMoO_3_(Lig)], Ref. [26] suggesting similar incorporation of {Mo^VI^O_3_} at the initial step of the reaction. Following the addition, the reaction was analyzed by NMR, IR, and UV-vis spectroscopy. Treatment of **2**(PF_6_) with one equivalent of (Et_4_N)_2_[MoO_4_] produced yellow-brown solution. The ^1^H NMR spectrum indicated the formation of several new products. The UV-vis spectrum of the crude product does not appear to contain any d-d transitions anticipated for Cu(II), suggesting that no Cu(I) oxidation takes place. Although we were not able to separate the products, IR spectroscopy provides a convenient tool to specifically interrogate the oxidation of isocyanide to isocyanate. An isocyanate is expected to exhibit an intense absorption between 2200 cm^−1^ and 2300 cm^−1^ (RN=C=O stretch). However, the IR spectrum of the crude product demonstrates a single sharp resonance around 2129 cm^−1^, consistent with the isocyanide stretch in aryl isocyanides (ArN≡C) [30]. As noted above, a stretching frequency of ~2130 cm^−1^ suggests either a free isocyanide, or an isocyanide coordinated mostly via σ-donation [28,29,30]. Such coordination would be expected for both Mo(VI) and Cu(I). No significant peaks above 2200 cm^−1^ were observed. 

The reactions of the phosphine complexes **3**(PF_6_) and **4**(PF_6_) with molybdate were investigated similarly. Treatment of the cold yellow solution of **3**(PF_6_) and **4**(PF_6_) with molybdate resulted in a slight color change to brown. In both cases, ^1^H NMR spectra of the products featured broad resonances. However, ^31^P NMR was more informative in both cases, allowing us to focus specifically on the phosphine transformation. Thus, the ^31^P NMR spectrum of the product of the reaction of **3**(PF_6_) with molybdate contains a single peak around −3 ppm (free *P*Ph_3_ is ~−6 ppm, *P*Ph_3_ in **3**(PF_6_) ~3 ppm, *P*(=O)Ph_3_ ~28 ppm) whereas the product of the reaction of **4**(PF_6_) with molybdate contains a peak around −46 ppm (free *P*Me_3_ is ~−62 ppm, *P*Me_3_ in **4**(PF_6_) ~−49 ppm, *P*(=O)Me_3_ 36 ppm). The findings above are consistent with coordinated phosphines and suggest that no oxidation of phosphines to the corresponding phosphine oxides takes place. 

**Figure 2:**
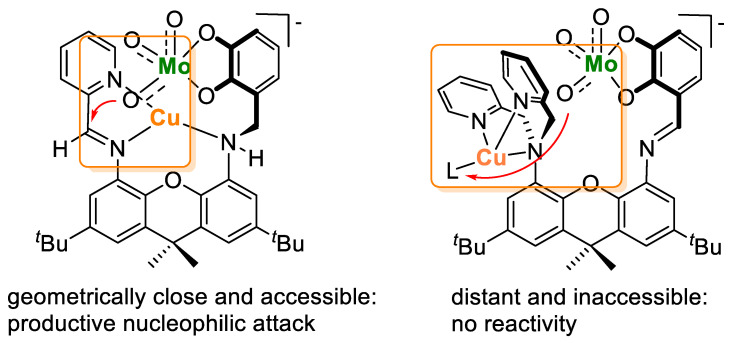
Comparison of the accessibility of Cu(I)-bound substrate to the nucleophilic attack by Mo-oxo in two different xanthene-based heterodinucleating systems [25,26].

The experiments above indicate the lack of in situ reactivity between Cu(I)-bound L (CNR, PR_3_) and [Mo^VI^O_3_]^2−^, and that is despite previously observed in situ nucleophilic attack of [Mo^VI^O_3_]^2−^ on the Cu(I)-bound imine [25]. We postulate that the lack of reactivity in the present system is due to the structural difference between the two heterodinucleating ligand systems (Figure 2). The iminopyridine system led to a relatively accessible three-coordinate Cu(I)-iminopyridine which, due to the coordination to the (xanthene-) amine, was positioned in the vicinity of Mo(VI)-oxo (Figure 2, left) [25]. In contrast, the coordination of the substrate to the dipicolylamine-coordinate Cu(I) leaves the metal four-coordinate, making it less accessible for attack. Furthermore, the coordination of the substrate L causes the displacement of the imine coordination which, combined with the more significant steric profile of dipicolylamine (vs. iminopyridine), leads to the *anti*-conformation of the Cu(I) fragment relative to the xanthene bridge (Figure 2, right). This factor is also likely to have a negative effect on reactivity.

### 2.3. Synthesis and Characterization of Mo_2_Cu_2_ Heterodinuclear Complex

**Scheme 3:**
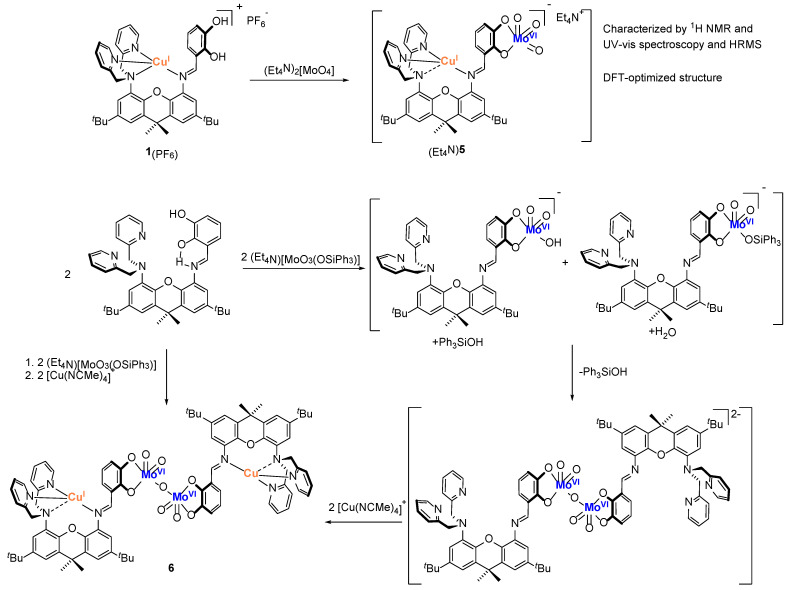
Top: Previously described formation of complex (Et_4_N)**5**. Bottom: Formation of complex **6** and possible reaction mechanism.

**Figure 3:**
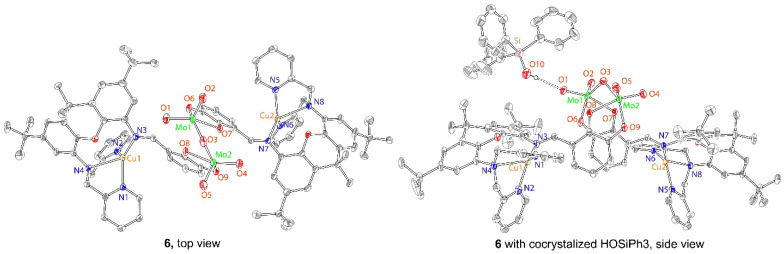
X-ray structure of **6**, 40% probability ellipsoids. Left: top view, without co-crystallized HOSiPh_3_ molecule. Right: side view, showing H-bonding between one of the basal oxos (O1) and the co-crystallized HOSiPh_3_ molecule. H atoms, co-crystallized acetonitrile molecules, and an alternative conformation for one of the tert-butyl groups were omitted for clarity. Selected bond distances: Mo1 O1 1.721(3), Mo1 O2 1.696(3), Mo1 O3 1.919(3), Mo2 O4 1.711(3), Mo2 O5 1.706(3), O1–O10 2.769(4) Å.

We have previously reported that the reaction of **1**(PF_6_) with molybdate formed red–brown (Et_4_N)[CuMoO_3_(Lig)] ((Et_4_N)**5**), which had limited stability in solution and therefore was characterized by ^1^H NMR, UV-vis spectroscopy, and HRMS; its DFT-optimized structure was in an agreement with spectroscopic data (Scheme 3). Our multiple attempts to crystallize (Et_4_N)**5** resulted in its decomposition. We hypothesized that the instability of (Et_4_N)**5** could be due to a highly reactive nature of catecholate-bound [Mo^VI^O_3_]^2−^, and thus decided to replace one of the oxo groups with siloxide. Treatment of (Et_4_N)[MoO_3_(OSiPh_3_)] with LigH_2_, followed by the addition of the resulting solution to [Cu(NCMe)_4_](PF_6_) resulted in an orange-brown solution. Recrystallization of the crude product from acetonitrile formed dark orange crystals of [Cu_2_Mo_2_O_4_(μ_2_-O)(Lig)_2_] (**6**). X-ray structure determination revealed **6** to be a tetranuclear complex containing two Mo(VI) and two Cu(I) centers (Figure 3 left). The complex is approximately *C*_2_-symmetric, albeit the symmetry is not crystallographic due, in part, to the presence of only one molecule of HOSiPh_3_ that is H-bonded to one of the equatorial oxos (Figure 3 right, see below for the discussion). The coordination geometry at each of the Cu(I) sites is similar to the geometry in **1**(PF_6_), with three relatively short copper-nitrogen (pyridine/imine) bond distances (ranging between 2.000(4) and 2.059(4) Å) and one very long copper-nitrogen (central amine) distance (2.432(4)/2.437(4) Å). The geometry at the molybdenum sites is approximately square-pyramidal, with the chelating catecholate occupying two of the four basal positions. The remaining basal positions and the apical positions are occupied by the oxo ligands, with bridging oxo located in the basal position. Related square-pyramidal [MO_3_(bdt)]^2−^/[MO_3−x_S_x_(bdt)]^2−^/[MO_2_(OSiR_3_)(bdt)]^2−^ (M = Mo, W; bdt = benzenedithiolate) were previously evaluated by Holm and coworkers as models for Xanthine Oxidoreductase [31,32,33]. The distance between catecholate oxygen and the second molybdenum (Mo1-O8 2.445(3)/Mo2-O7 2.452(3) Å) is likely too long for a bond. Bridging oxo demonstrates a relatively long molybdenum-oxo bond distance of 1.918(4)/1.919(4) Å, compared with 1.696(3)–1.721(4) Å for the terminal oxos. It is noted the relative metals disposition in each of the [Mo-Cu] “halves” is highly reminiscent of the calculated structure of [CuMoO_3_(Lig)]^+^ (**5^+^**) [26].

As mentioned above, the complex is co-crystallized with one molecule of Ph_3_SiOH that is hydrogen-bonded to one of the equatorial oxos (Figure 3, right). The presence of one equivalent of triphenylsilanol per two molybdenum ions suggests the dimerization mechanism presented in Scheme 3. Unlike [MoO_4_]^2−^, [MoO_3_(OSiPh_3_)]^−^ contains two different protonation sites. Thus, it is feasible that the acid-base reaction of catechol with [MoO_3_(OSiPh_3_)]^−^ leads to the formation of siloxo-terminated and hydroxo-terminated products. Subsequent condensation of these complexes results in Mo-(μ_2_-O)-Mo complex which produces the final product **6** upon the incorporation of two Cu(I) ions.

Complex **6** was also characterized in solution, by ^1^H NMR spectroscopy and mass spectrometry. Although the spectrum in CD_3_CN demonstrated only broad peaks, the spectrum in DMF-d_7_ featured relatively sharp resonances consistent with the overall structure of **6**. Additional support for the tetranuclear structure of **6** comes from the observation of the peak corresponding to the molecular ion (**6**^+^) at *m/z* = 1706.3350 (expected *m/z* = 1706.3273), albeit at low intensity.

## 3. Summary and Conclusions

Towards the development of the functional model of Mo-Cu CODH, we have previously reported the synthesis of Cu(I)-Mo(VI) complex with a xanthene-bridged heterodinucleating ligand LigH_2_ featuring dipycolylamine chelate for Cu(I) and catecholate for Mo(VI). However, the complex had only limited stability in solution, and could not be obtained in a solid state; therefore, its subsequent reactivity was not investigated. In the present work, we targeted: (1) the in situ reactivity studies combining Lig, Cu(I), Mo(VI), and a potentially oxidizable ligand L (isocyanide, phosphine, cyanide), and (2) a more stable heterodinuclear Mo^VI^Cu^I^ complex which could be isolated and structurally characterized. Towards the first goal, we have prepared a series of Lig-supported Cu(I)-L complexes, treated them with [MoO_4_]^2−^, and explored the nature of the product (L vs. L=O) using the corresponding spectroscopy. For both phosphine and isocyanide substrates, no oxidation was observed. We propose that the lack of oxidation reactivity can be attributed (at least in part) to the difficulty in accessing a Cu(I)-bound substrate, which is removed from the Mo site and is obscured by the steric congestion at the Cu site. Toward our second goal, we explored the reactivity of the [MoO_3_(OSiPh_3_)]^−^ precursor, which is expected to produce more stable Mo(VI) complexes. The incorporation of both Mo(VI) and Cu(I) metals within the ligand framework produced a tetranuclear Mo^VI^_2_Cu^I^_2_ product [Cu_2_Mo_2_O_4_(μ_2_-O)(Lig)_2_], in which two heterodinuclear {Mo^VI^Cu^I^(Lig^1^)} fragments were further linked by a μ_2_-oxo ligand at Mo. The formation of the tetranuclear product was accompanied by the release of silanol directly observed in the crystal. The presence of one equivalent of Ph_3_SiOH per two molybdenum centers suggests a dimerization mechanism, which involves the reaction of Mo-OSiPh3 with Mo-OH intermediate to form Mo-O-Mo and Ph_3_SiOH. As in the previously reported DFT-optimized structure of the heterodinuclear [Mo^VI^Cu^I^(Lig^1^)] complex, the metals were relatively far from each other (~7 Å away), which is consistent with the lack of heterobimetallic reactivity. Our future studies in this project will focus on heterodinucleating ligands in which the Cu(I) site is anchored to the Mo site by the nearby imino(phenolate) or amino(phenolate) donor.

## 4. Materials and Methods

### 4.1. General

All reactions involving air-sensitive materials were carried out in a nitrogen-filled glovebox. 2,7-Di-tert-butyl-9,9-dimethyl-9H-xanthene-4,5-diamine, (E)-3-(((5-(bis(pyridin-2-ylmethyl) amino)-2,7-di-tert-butyl-9,9-dimethyl-9H-xanthen-4-yl)imino)methyl)benzene-1,2-diol, tetraethylammonium molybdate, **1**(PF_6_), and (Et_4_N)**3** were synthesized using previously published procedures [26,34,35,36]. 2,6-Dimethylphenyl isocyanide, triphenylphosphine, and trimethylphosphine, were purchased from Sigma and used as received. Tetrakis(acetonitrile)copper(I) hexafluorophosphate was purchased from Strem and used as received. All non-deuterated solvents were purchased from Aldrich and were of HPLC grade. The non-deuterated solvents were purified using an MBraun solvent purification system. Dichloromethane-d_2_ and acetonitrile-d_3_ were purchased from Cambridge Isotope Laboratories. All solvents were stored over 3 Å molecular sieves. Compounds were generally characterized by ^1^H and ^13^C NMR spectroscopy (including 2D techniques such as ^1^H-^1^H COSY and HMBC) and high-resolution mass spectrometry. Selected compounds were characterized by X-ray crystallography. Compounds characterization was carried out at the Lumigen Instrument Center at Wayne State University. NMR spectra of metal complexes were recorded on an Agilent DD2-600 MHz spectrometer, and an Agilent 400 MHz spectrometer in CD_3_CN and DMF at room temperature. Chemical shifts and coupling constants (*J*) were reported in parts per million (*δ*) and Hertz, respectively. Detailed assignments of the signals in ^1^H NMR are given in the ESI. High-resolution mass spectra of the metal complexes (unless otherwise stated) were collected on a Thermofisher Scientific LTQ Orbitrap XL mass spectrometer. The MS survey scan was set from 200 to 2000 *m/z*. The resolution was set to 60,000. In all cases, only one microscan was used in the analysis. 

### 4.2. Synthetic Procedures and Compounds Characterization

Preparation of [Cu(CN(2,6-Me_2_C_6_H_3_))(LigH_2_)](PF_6_) (**2**(PF_6_). A 3 mL solution of 2,6-Dimethylphenyl isocyanide (3.80 mg, 0.029 mmol, 1.0 equiv.) in CH_3_CN was added dropwise to a stirring 5 mL solution of complex [Cu(LH_2_)](PF_6_)] (25 mg, 0.029 mmol, 1.0 equiv) in CH_3_CN. The reaction mixture was stirred for 1 h, upon which the volatiles were removed in vacuo. This solid was purified by recrystallization from CH_2_Cl_2_/diethyl ether, which yielded orange-yellow crystals of [Cu(CN(2,6-Me_2_C_6_H_3_))(LigH_2_)](PF_6_) (26 mg, 0.028 mmol, 90%). ^1^H NMR (CD_3_CN, 400 MHz) δ 8.79 (s, 1H, imine-H), 8.72 (d, ^3^*J*_HH_ = 5.1 Hz, 2H, α-H on pyridine), 7.70 (s, 1H, ortho-H on pyridinyl xanthene side), 7.66 (t, ^3^*J*_HH_ = 7.8 Hz, 2H, γ-H on pyridine), 7.50 (s, 1H, para-H on pyridinyl xanthene side), 7.43 (s, 1H, para-H on catechol xanthene side), 7.37 (t, ^3^*J*_HH_ = 4.8 Hz, 2H, β-H on pyridine),7.31 (t, ^3^*J*_HH_ = 7.1 Hz, 1H, para-H on isocyanide), 7.20 (m, 2H, meta-H on isocyanide), 7.18 (s, 1H, ortho-H on catechol xanthene side), 7.12 (d, ^3^*J*_HH_ = 7.6 Hz, 1H, 4-H on catechol), 7.07 (d, ^3^*J*_HH_ = 7.8 Hz, 2H, β’-H on pyridine), 6.93 (d, ^3^*J*_HH_ = 7.9 Hz, 1H, 6-H on catechol), 6.88 (t, ^3^*J*_HH_ = 7.7 Hz, 1H, 5-H on catechol), 4.67 (s, 4H, methylene-H), 2.32 (s, 6H, methyl-H on isocyanide), 1.69 (s, 6H, methyl-H), 1.37 (s, 9H, tert-butyl-H on catechol xanthene side), 1.14 (s, 9H, tert-butyl-H on pyridinyl xanthene side) ppm. ^13^C{^1^H} NMR (CD_2_Cl_2_,150 MHz) δ 165.47, 157.26, 149.37, 149.30, 146.97, 145.57, 145.42, 143.94, 140.00, 138.59, 136.60, 135.28, 134.42, 130.98, 129.77, 129.75, 128.11, 124.26, 124.07, 123.93, 123.27, 122.25, 121.75, 119.14, 118.97, 118.32, 116.05, 58.76, 34.59, 34.30, 32.42, 30.62, 30.47, 17.96. HRMS (ESI^+^) *m/z* 848.3577 (calculated *m/z* for [Cu(CN(2,6-Me_2_C_6_H_3_))(LigH_2_)]^+^ 848.3595). IR (ATR) 2137 (C≡NAr) cm^−1^.

Reaction of [Cu(CN(2,6-Me_2_C_6_H_3_))(LigH_2_)](PF_6_) (**2**(PF_6_)) with (NEt_4_)_2_[MoO_4_]. A 3 mL solution of tetraethylammonium molybdate (NEt_4_)_2_[MoO_4_] (8.72 mg, 0.020 mmol, 1.0 equiv) in CH_3_CN and a 3 mL solution of [Cu(LH_2_)(ArNC)](PF_6_) (20 mg, 0.020 mmol, 1.0 equiv.) in CH_3_CN were prepared and cooled to −33 °C. The solution of cold [Cu(LH_2_)(ArNC)](PF_6_) was then added dropwise to a stirring solution of cold (NEt_4_)_2_[MoO_4_]. The reaction mixture was stirred for 1hr, after which the volatiles were removed in vacuo to obtain an orang-yellow solid. The crude product was characterized by ^1^H NMR and IR.

Preparation of [Cu(PMe_3_)(LigH_2_)](PF_6_) (**4**(PF_6_)). A 6 µL (0.057 mmol, 1.0 equiv) solution of trimethylphosphine, a 5 mL solution of complex [Cu(LH_2_)](PF_6_)] (50 mg, 0.057 mmol, 1.0 equiv) in DCM were prepared and cooled to −33 °C. The solution of cold trimethylphosphine was then added dropwise to a stirring solution of cold[Cu(LH_2_)](PF_6_)]. The reaction mixture was stirred for 1 h, upon which the volatiles were removed in vacuo. The product [Cu(LH_2_)(ArNPMe_3_)](PF_6_) was obtained as an orange solid. (51.50 mg, 0.055 mmol, 95%).^1^H NMR (CD_3_CN, 400 MHz) δ 13.93 (br, s, 1H, catechol-OH), 8.91 (s, 1H, imine-H), 8.66 (d, ^3^*J*_HH_ = 5.0 Hz, 2H, α-H on pyridine), 7.57 (t, ^3^*J*_HH_ = 7.7 Hz, 2H, γ-H on pyridine), 7.54 (s, 1H, ortho-H on pyridinyl xanthene side), 7.46 (s, 2H, para-H on pyridinyl xanthene side, para-H on catechol xanthene side), 7.36 (t, ^3^*J*_HH_ = 6.4 Hz, 2H, β-H on pyridine), 7.32 (s, 1H, ortho-H on catechol xanthene side), 7.19 (d, ^3^*J*_HH_ = 7.4 Hz, 1H, 4-H on catechol), 6.92 (m, 3H, β’-H on pyridine, 6-H on catechol), 6.90 (m, 1H, 5-H on catechol), 6.15 (br, 1H, catechol-OH) 4.66 (s, 4H, methylene-H), 1.72 (s, 6H, methyl-H), 1.41 (s, 9H, tert-butyl-H on catechol xanthene side), 1.29 (s, 6H, methyl-H on isocyanide) 1.15 (s, 9H, tert-butyl-H on pyridinyl xanthene side) ppm. ^13^C{^1^H} NMR (CD_2_Cl_2_,150 MHz) δ 165.44, 157.57, 149.51, 149.29, 146.99, 145.44, 145.39, 145.02, 140.23, 138.09, 136.34, 134.88, 131.12, 129.82, 125.66, 123.97, 123.73, 123.27, 122.92, 121.90, 119.17, 119.06, 118.26, 115.75, 67.30, 65.27, 58.71, 54.31, 34.65, 34.59, 34.18, 32.45, 30.65, 30.62, 25.26, 14.63. HRMS (ESI+) *m/z* 793.3295 (calculated *m/z* for [Cu(LH_2_)(ArNPMe_3_)^+^] 793.3302). ^31^P NMR (600 MHz) δ −49.0 ppm.

Reaction of [Cu(PMe_3_)(LigH_2_)](PF_6_) (**4**(PF_6_)) with (NEt_4_)_2_[MoO_4_]. A 3 mL solution of tetraethylammonium molybdate (NEt_4_)_2_[MoO_4_] (8.95 mg, 0.021 mmol, 1.0 equiv) in CH_3_CN and a 3 mL solution of [Cu(LH_2_)(ArNPMe_3_)](PF_6_) (20 mg, 0.021 mmol, 1.0 equiv.) in CH_3_CN were prepared and cooled to −33 °C. The solution of cold [Cu(LH_2_)(ArNPMe_3_)](PF_6_) was then added dropwise to a stirring solution of cold (NEt_4_)_2_[MoO_4_]. The reaction mixture was stirred for 1hr, after which the volatiles were removed in vacuo to obtain an orange solid.

Reaction of [Cu(PPh_3_)(LigH_2_)](PF_6_) (**3**(PF_6_)) with (NEt_4_)_2_[MoO_4_]. A 3 mL solution of tetraethylammonium molybdate (NEt_4_)_2_[MoO_4_] (7.38 mg, 0.017 mmol, 1.0 equiv) in CH_3_CN and a 3 mL solution of [Cu(LH_2_)(PPh_3_)](PF_6_). (20 mg, 0.017 mmol, 1.0 equiv.) in CH_3_CN were prepared and cooled to −33 °C. The solution of cold [Cu(LH_2_)(PPh_3_)](PF_6_) (2(PF_6_)). was then added dropwise to a stirring solution of cold (NEt_4_)_2_[MoO_4_]. The reaction mixture was stirred for 1hr, after which the volatiles were removed in vacuo to obtain an orange solid.

Preparation of [Cu_2_Mo_2_O_4_(μ_2_-O)(Lig)_2_] (**6**)**.** A 3 mL solution of LH_2_ (15 mg, 0.022 mmol, 1.0 equiv) in CH_3_CN and a 3 mL solution of (Et_4_N)[MoO_3_(OSiPh_3_)] (12.59 mg, 0.022 mmol, 1.0 equiv.) in CH_3_CN were prepared and cooled to −33 °C. The solution of cold LH_2_ was then added dropwise to a stirring solution of cold (Et_4_N)[MoO_3_(OSiPh_3_)]. The reaction mixture was stirred for 15 min and 3 mL solution of tetrakis(acetonitrile)copper(I) hexafluorophosphate [Cu(NCMe)_4_](PF_6_) (8.53 mg, 0.022 mmol, 1.0 equiv.) was added. The reaction mixture was stirred for 30 min, after which the volatiles were removed in vacuo to produce a dark orange solid and crude product was recrystallized using CH_3_CN to give orange crystals of **6** (13.6 mg, 0.008 mmol, 70%, co-crystallized with HOSiPh_3_). To remove HOSiPh_3_, **6** was briefly washed with CH_3_CN and dissolved in DMF. ^1^H NMR (DMF, 400 MHz) δ 10.44 (s, 1H, imine-H), 8.88 (s, 1H, 4-H on catechol), 8.15 (s, 1H, ortho-H on pyridinyl xanthene side), 7.89 (m, 3H, para-H on pyridinyl xanthene side, β-H on pyridine), 7.67 (m, 1H, para-H on catechol xanthene side), 7.58 (m, 2H, α-H on pyridine), 7.45 (m, 2H, β-H on pyridine), 7.39 (s, 1H, ortho-H on catechol xanthene side), 7.30 (m, 2H, γ-H on pyridine), 6.55 (s, 1H, 6-H on catechol, 6.39 (m, 1H, 5-H on catechol), 4.88 (br s, 2H, methylene-H) 4.30 (br s, 2H, methylene-H), 1.56 (s, 6H, Me), 1.37 (s, 18H, t-Bu) ppm. HRMS (ESI^+^) *m/z* 1706.3350 (calculated *m/z* for [Cu_2_Mo_2_O_4_(μ_2_-O)(Lig)_2_]^+^ 1706.3267). 

### 4.3. X-ray Crystallographic Details

All crystal structures were collected on a Bruker D8 Venture diffractometer with kappa geometry, an Incoatec IµS micro-focus X-ray source (Mo K_α_ radiation), and a multilayer mirror for monochromatization. The diffraction intensities were measured using a Bruker Photon III CPAD area detector. Data were acquired at 100 K with an Oxford 800 Cryostream low-temperature apparatus. The data were processed using APEX4 software (v.2022.10-0, Bruker, Madison, USA). The structures were solved by Intrinsic Phasing using ShelXT [37] and refined with ShelXL [38] using Olex2 [39]. Hydrogen atoms were placed in calculated positions using a standard riding model and refined isotropically; all other atoms were refined anisotropically. The structure of **2**(PF_6_) contained one co-crystallized molecule of diethyl ether. The structure of **6** contained one co-crystallized molecule of HOSiPh_3_ and six co-crystallized molecules of acetonitrile. In addition, one of the tert-butyl groups of **6** was found to be disordered over two positions. The main refinement parameters are listed in Table 1. The structures were uploaded in the CCDC under the following numbers: 2252288–2252289.

## Figures and Tables

**Table 1:** Crystal and structure refinement data.

	2(PF_6_)	6
Formula	C_51_H_55_CuN_5_O_3_•PF_6_•C_4_H_10_O	C_84_H_88_Cu_2_Mo_2_N_8_O_11_•C_18_H_16_OSi•6(C_2_H_3_N)
Fw, g/mol	1068.670	2227.30
temperature	100 K	100 K
X-ray source	MoKα	MoKα
cryst syst	triclinic	monoclinic
space group	*P-1*	*P2_1_/n*
color	yellow	orange
Z	2	4
a, Å	13.6310(5)	20.546(5)
b, Å	14.4630(6)	21.591(7)
c, Å	15.8288(6)	25.679(7)
α, deg	112.847(1)	90.00
β, deg	92.606(1)	109.253(6)
γ, deg	109.383(1)	90.00
V, A^3^	2657.65(18)	10754(5)
d_calcd_, g/cm^3^	1.335	1.376
μ, mm^−1^	0.512	0.696
2θ, deg	55.00	55.96
R_1_^a^ (all data)	0.0428	0.0996
wR_2_^b^ (all data)	0.1011	0.1977
R_1_^a^ [(I > 2σ)]	0.0372	0.0682
wR_2_^b^ [(I > 2σ)]	0.0969	0.1760
GOF ^c^	1.040	1.022

^a^ R_1_ = Σ||F_o_ − |F_c_||/|F_o_|. ^b^ wR_2_ = ((w(F_o_^2^ − F_c_^2^)^2^)/(w(F_o_^2^)^2^))^1/2^. ^c^ GOF = (w(F_o_^2^ − F_c_^2^)^2^/(n − p))^1/2^ where n is the number of data and p is the number of parameters refined.

## Data Availability

Information about Supplementary Materials is provided above in the “Supplementary Materials” statement.

## Supplementary Materials

The following supporting information can be downloaded at: https://www.mdpi.com/article/10.3390/molecules28083644/s1, General experimental details, synthetic procedures, crystallographic, NMR, IR, and mass spectra. Figure S1. ^1^H NMR spectrum of [Cu(CN(2,6-Me_2_C_6_H_3_))(LigH_2_)](PF_6_) (**2**(PF_6_)) (CD_3_CN, 400 MHz). Figure S2. ^1^H-^1^H COSY NMR spectrum of [Cu(CN(2,6-Me_2_C_6_H_3_))(LigH_2_)](PF_6_) (**2**(PF_6_)) (CD_3_CN, 400 MHz). Figure S3. ^13^C NMR spectrum of [Cu(CN(2,6-Me_2_C_6_H_3_))(LigH_2_)](PF_6_) (**2**(PF_6_)) (CD_3_CN, 150 MHz). Figure S4. ^1^H NMR spectrum of the reaction between [Cu(CN(2,6-Me_2_C_6_H_3_))(LigH_2_)](PF_6_) (**2**(PF_6_)) and (NEt_4_)2[MoO_4_] (CD_3_CN, 400 MHz). Figure S5. ^1^H NMR spectrum of [Cu(PMe_3_)(LigH_2_)](PF_6_) (**4**(PF_6_)) (CD_3_CN, 400 MHz). Figure S6. ^1^H-^1^H COSY NMR spectrum of [Cu(PMe_3_)(LigH_2_)](PF_6_) (**4**(PF_6_)) (CD_3_CN 400 MHz). Figure S7. ^13^C NMR spectrum of [Cu(PMe_3_)(LigH_2_)](PF_6_) (**4**(PF_6_)) (CD_3_CN 150 MHz). Figure S8. ^31^P NMR spectrum of [Cu(PMe_3_)(LigH_2_)](PF_6_) (**4**(PF_6_)) (CD_3_CN 400 MHz). Figure S9. ^1^H NMR spectrum of the reaction between [Cu(PMe_3_)(LigH_2_)](PF_6_) (**4**(PF_6_)) with (NEt_4_)2[MoO_4_] (CD_3_CN, 400 MHz). Figure S10. ^31^P NMR spectrum of the reaction between [Cu(PMe_3_)(LigH_2_)](PF_6_) (**4**(PF_6_)) with (NEt_4_)2[MoO_4_] (CD_3_CN, 400 MHz). Figure S11. ^1^H NMR spectrum of the reaction between [Cu(PPh_3_)(LigH_2_)](PF_6_) (**3**(PF_6_) and (NEt_4_)2[MoO_4_] (CD_3_CN, 400 MHz). Figure S12. ^31^P NMR spectrum of the between [Cu(PPh_3_)(LigH_2_)](PF_6_) (**3**(PF_6_) and (NEt_4_)2[MoO_4_] (CD_3_CN, 400 MHz). Figure S13. ^1^H NMR spectrum of the reaction between **1**(PF_6_) and NaCN (CD_3_CN, 400 MHz). Figure S14. ^1^H NMR spectrum of the reaction between **1**(PF_6_) and styrene (CD_2_Cl_2_, 400 MHz). Figure S15. ^1^H NMR spectrum of [Cu_2_Mo_2_O_4_(μ_2_-O)(Lig)_2_] (**6**) (CD_3_CN, 400 MHz). Figure S16. ^1^H NMR spectrum of [Cu_2_Mo_2_O_4_(μ_2_-O)(Lig)_2_] (**6**) (DMF-d_7_, 400 MHz). Figure S17. ^1^H-^1^H COSY NMR spectrum of [Cu_2_Mo_2_O_4_(μ_2_-O)(Lig)_2_] (**6**) (DMF-d_7_, 400 MHz). Figure S18. High-resolution mass spectrum of [Cu(CN(2,6-Me_2_C_6_H_3_))(LigH_2_)]+ (**2**+). Figure S19. High-resolution mass spectrum of [Cu(PMe_3_)(LigH_2_)]+ (**3**+). Figure S20. High-resolution mass spectrum of [Cu_2_Mo_2_O_4_(μ_2_-O)(Lig)_2_]+ (**6**+). Figure S21. IR spectrum of [Cu(CN(2,6-Me_2_C_6_H_3_))(LigH_2_)](PF_6_) (**2**(PF6) (isocyanide CN stretch region). Figure S22. IR spectrum of the reaction product between [Cu(CN(2,6-Me_2_C_6_H_3_))(LigH_2_)](PF_6_) (**2**(PF_6_) and (NEt_4_)_2_[MoO_4_] (isocyanide CN stretch region).
