# Supplementary material for: Studies Relevant to the Functional Model of Mo-Cu CODH: In Situ Reactions of Cu(I)-L Complexes with Mo(VI) and Synthesis of Stable Structurally Characterized Heterotetranuclear MoVI2CuI2 Complex
Source: Molecules. 2023 Apr 21;28(8):3644. doi: 10.3390/molecules28083644 (PMC10143188; doi:10.3390/molecules28083644)
Supplement: Supplementary file 1 [file molecules-28-03644-s001.zip › SI/SI.pdf]

Electronic Supplementary Information

For

Studies Relevant to the Functional Model of Mo-Cu CODH: In-situ Reactions of Cu(I)-L Complexes with Mo(VI) and Synthesis of Stable Structurally Characterized Heterotetranuclear $Mo^{VI}_2Cu^{I}_2$ Complex

Umesh I. Kaluarachchige Don,[a] Ahmad S. Almaat,[a] Cassandra L. Ward,[b] Stanislav Groysman[a]*

[a]Department of Chemistry, Wayne State University, 5101 Cass Ave. Detroit MI 48202.

[b]Lumigen Instrument Center, Wayne State University, 5101 Cass Avenue, Detroit, Michigan 48202, United States.

E-mail: groysman@wayne.edu

Table of Contents

1. NMR spectra	3
2. Mass spectra	20
3. IR spectra	23

1. NMR spectra

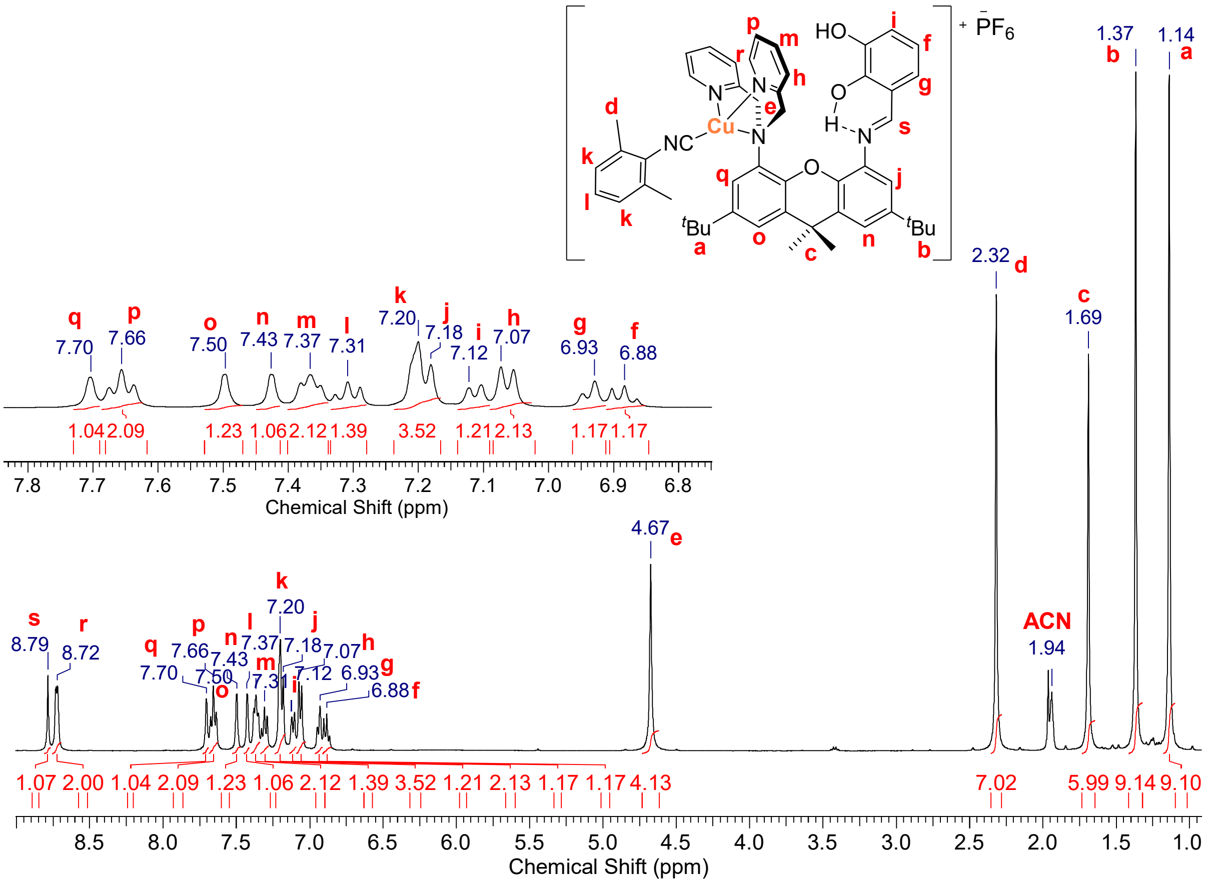

Figure S1. ^{1}H NMR spectrum of [Cu(CN(2,6-$Me_2C_6H_3$))($LigH_2$)](PF_6) (**2**(PF_6)) (CD_3CN, 400 MHz).

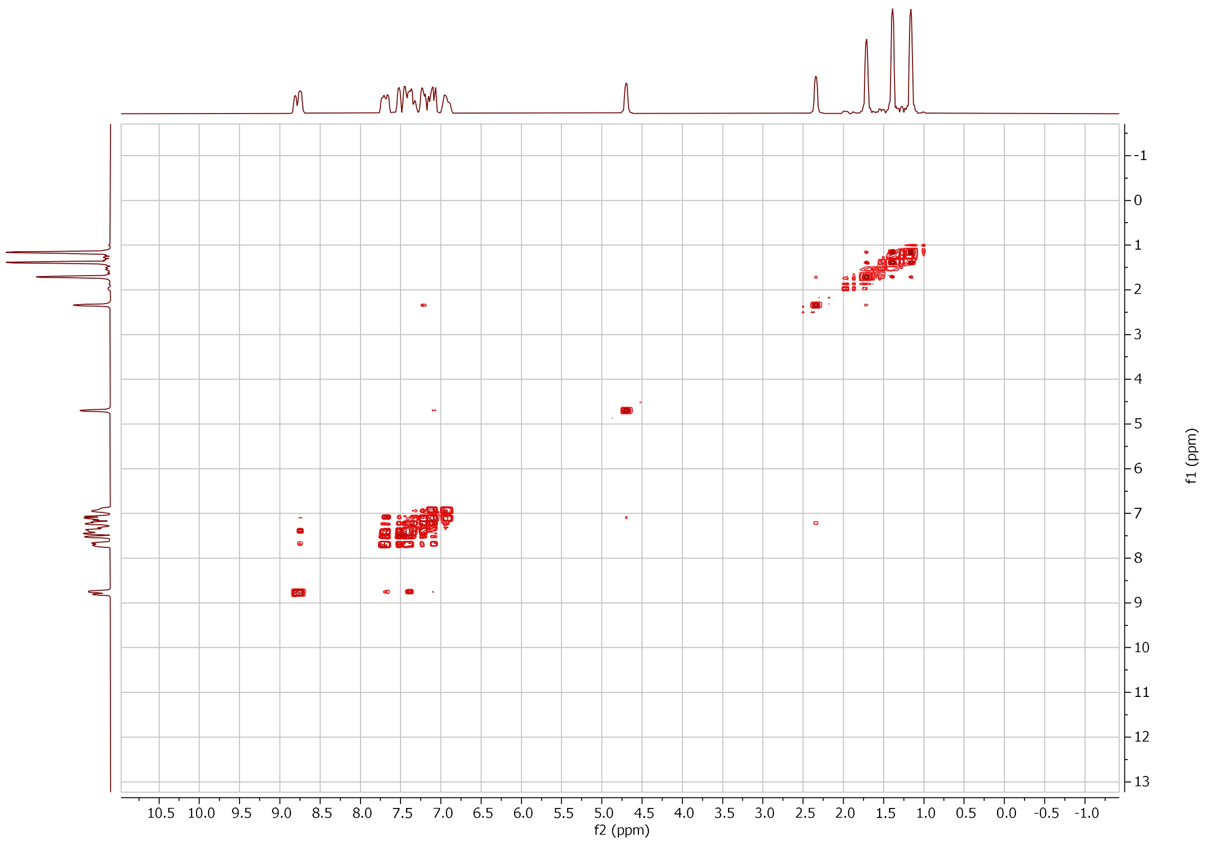

Figure S2. ^1H-^1H COSY NMR spectrum of [Cu(CN(2,6-$Me_2C_6H_3$))(LigH_2)](PF_6) (**2**(PF_6)) (CD_3CN, 400 MHz).

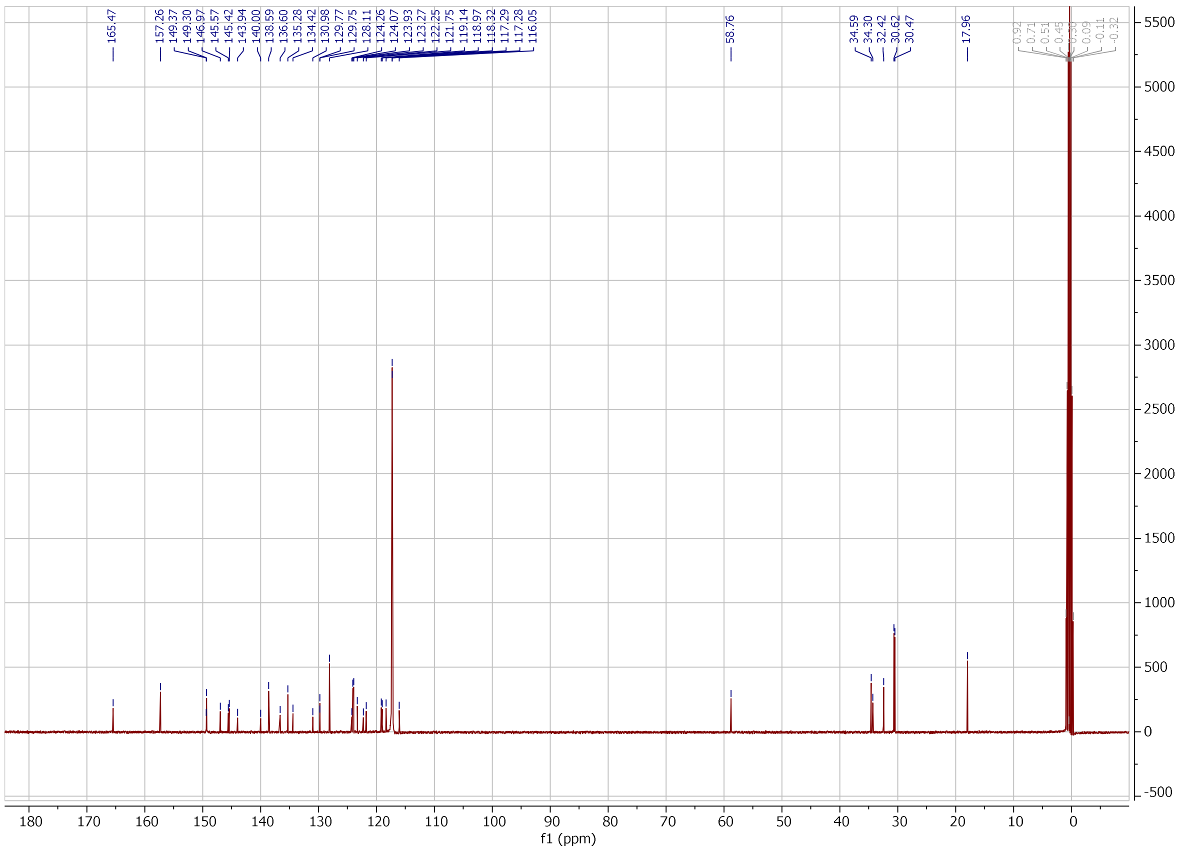

Figure S3. ^{13}C NMR spectrum of $[Cu(CN(2,6-Me_2C_6H_3))(LigH_2)](PF_6)$ (**2**(PF_6)) (CD_3CN, 150 MHz).

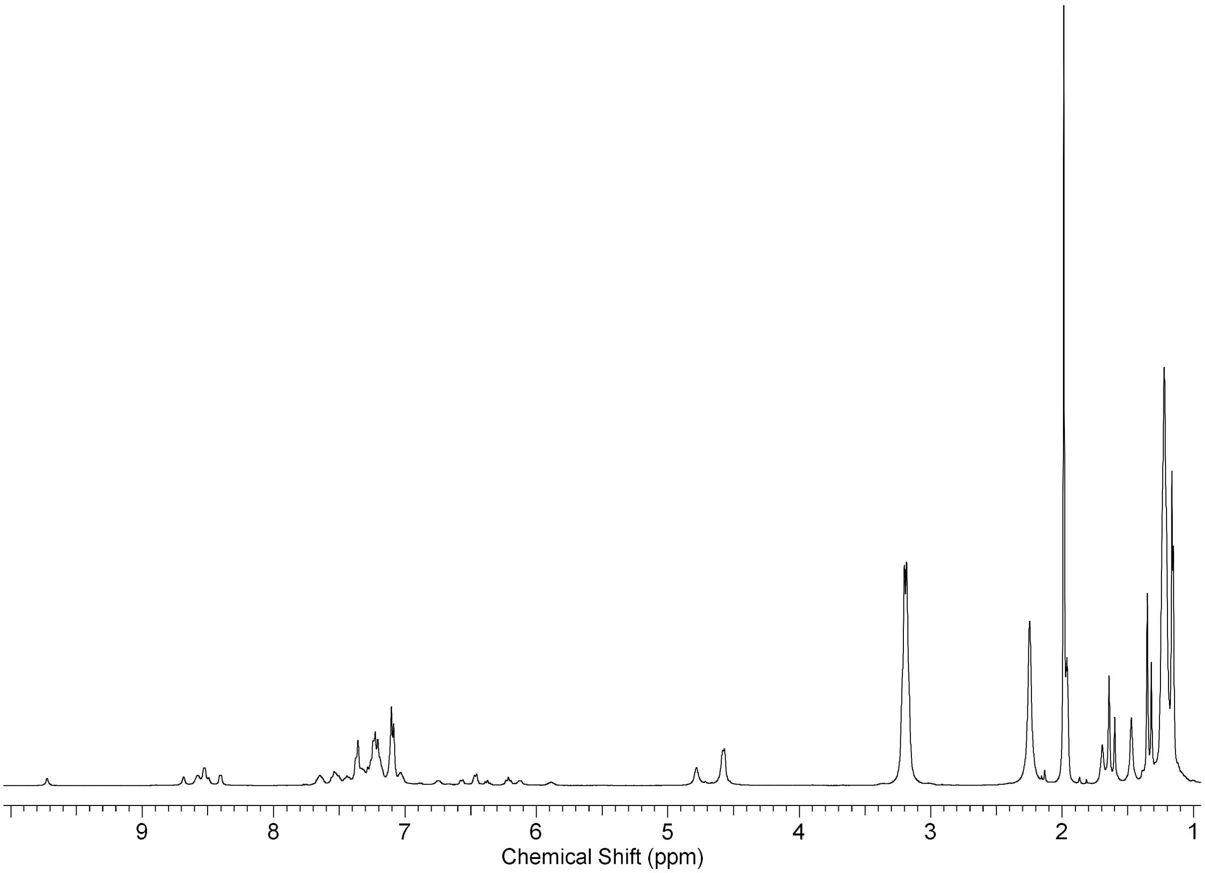

Figure S4. ^{1}H NMR spectrum of the reaction between $[Cu(CN(2,6\text{-}Me_2C_6H_3))(LigH_2)](PF_6)$ (**2**(PF_6)) and $(NEt_4)_2[MoO_4]$ (CD_3CN, 400 MHz).

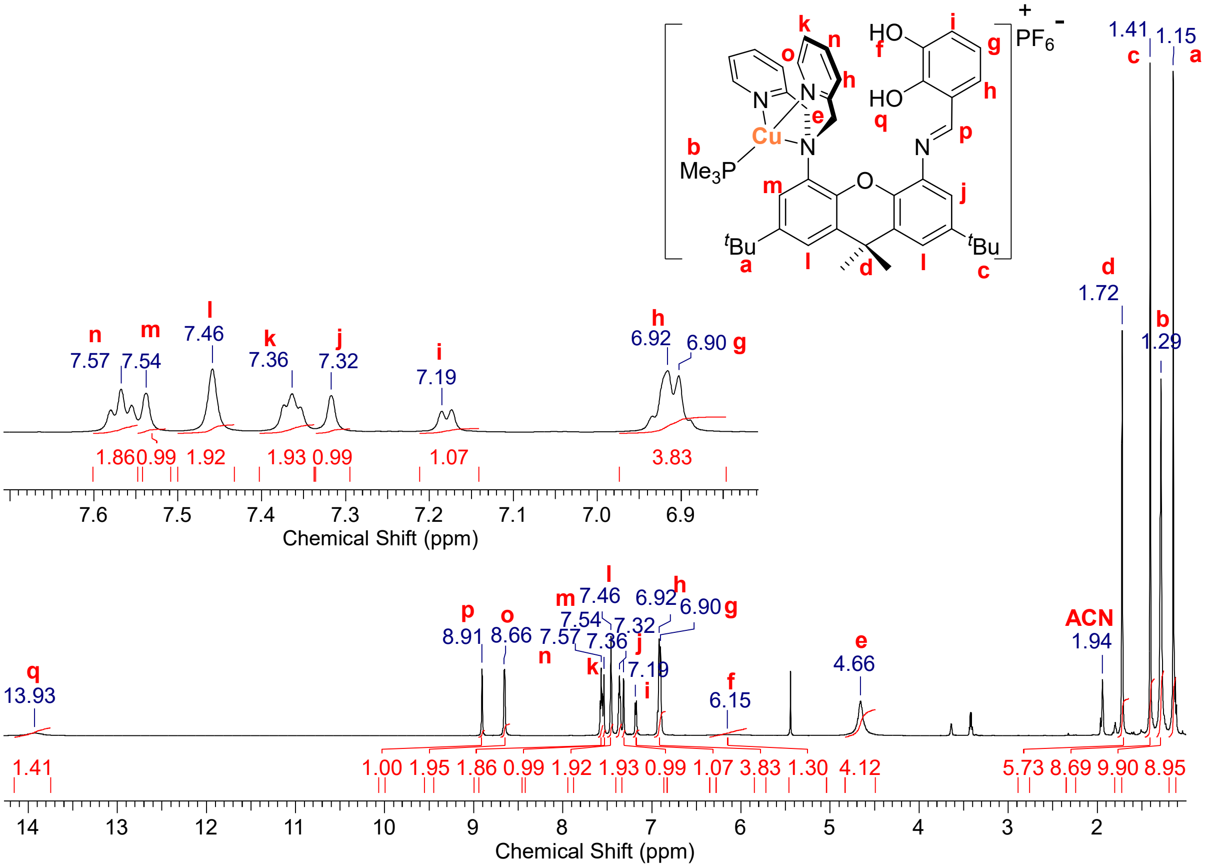

Figure S5. ^{1}H NMR spectrum of $[Cu(PMe_3)(LigH_2)](PF_6)$ (**4**(PF_6)) (CD_3CN, 400 MHz).

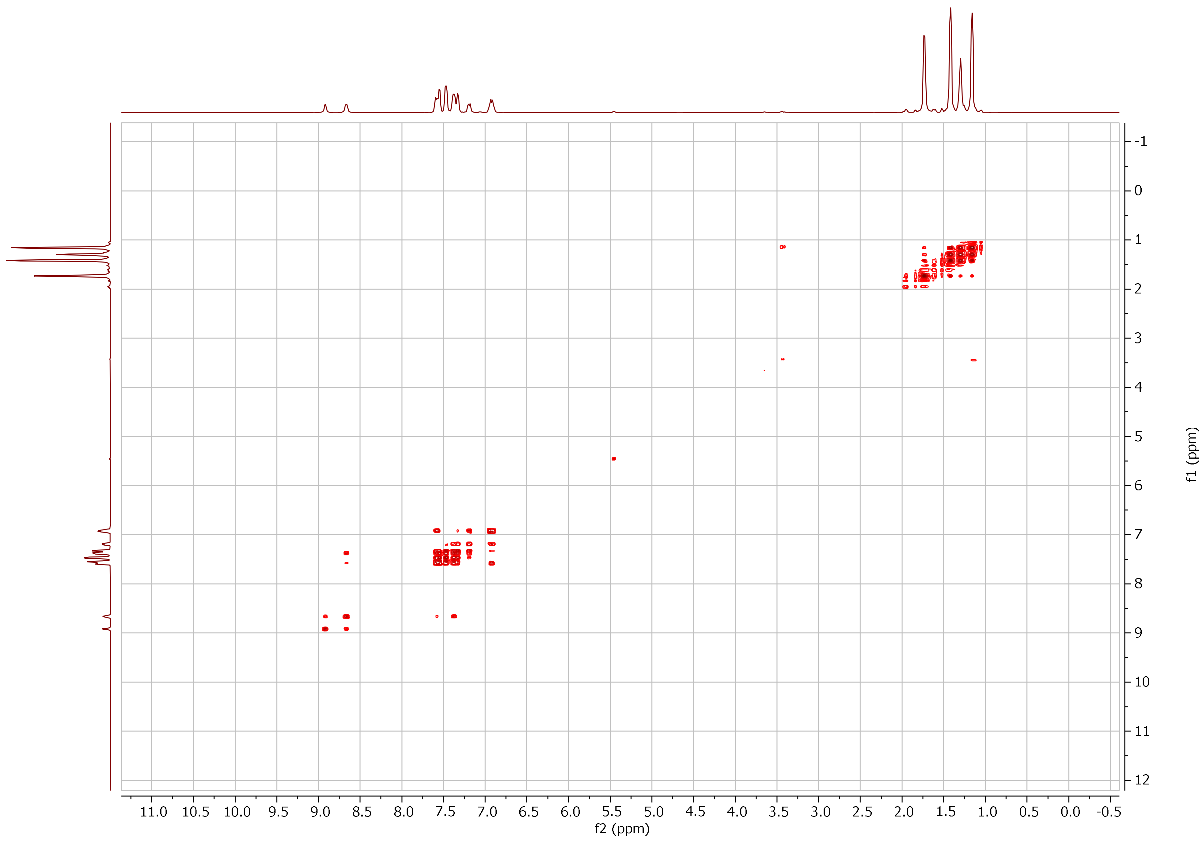

Figure S6. ^{1}H-^{1}H COSY NMR spectrum of $[Cu(PMe_3)(LigH_2)](PF_6)$ (**4**(PF_6)) (CD_3CN, 400 MHz)

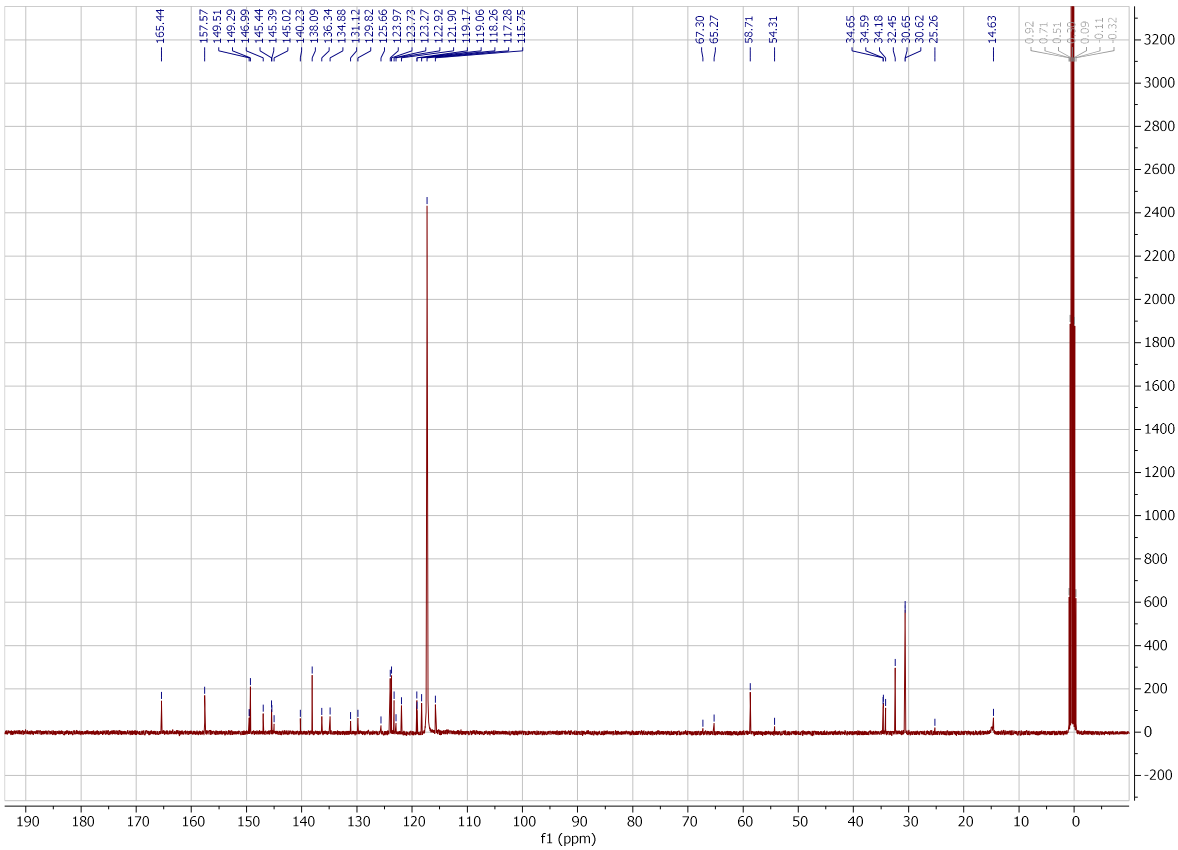

Figure S7. ^{13}C NMR spectrum of [Cu(PMe$_3$)(LigH$_2$)](PF$_6$) (**4**(PF$_6$)) (CD$_3$CN, 150 MHz)

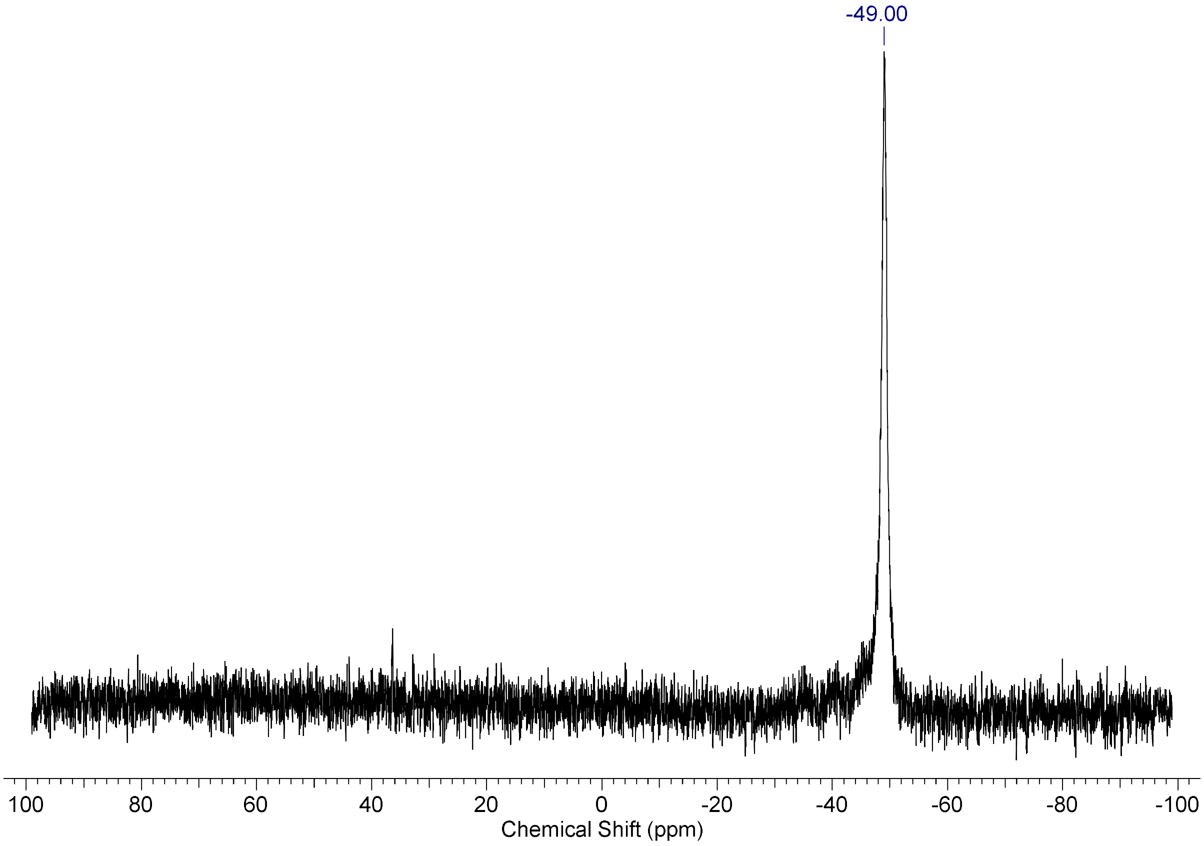

Figure S8. ^{31}P NMR spectrum of [Cu(PMe$_3$)(LigH$_2$)](PF$_6$) (**4**(PF$_6$)) (CD$_3$CN, 400 MHz).

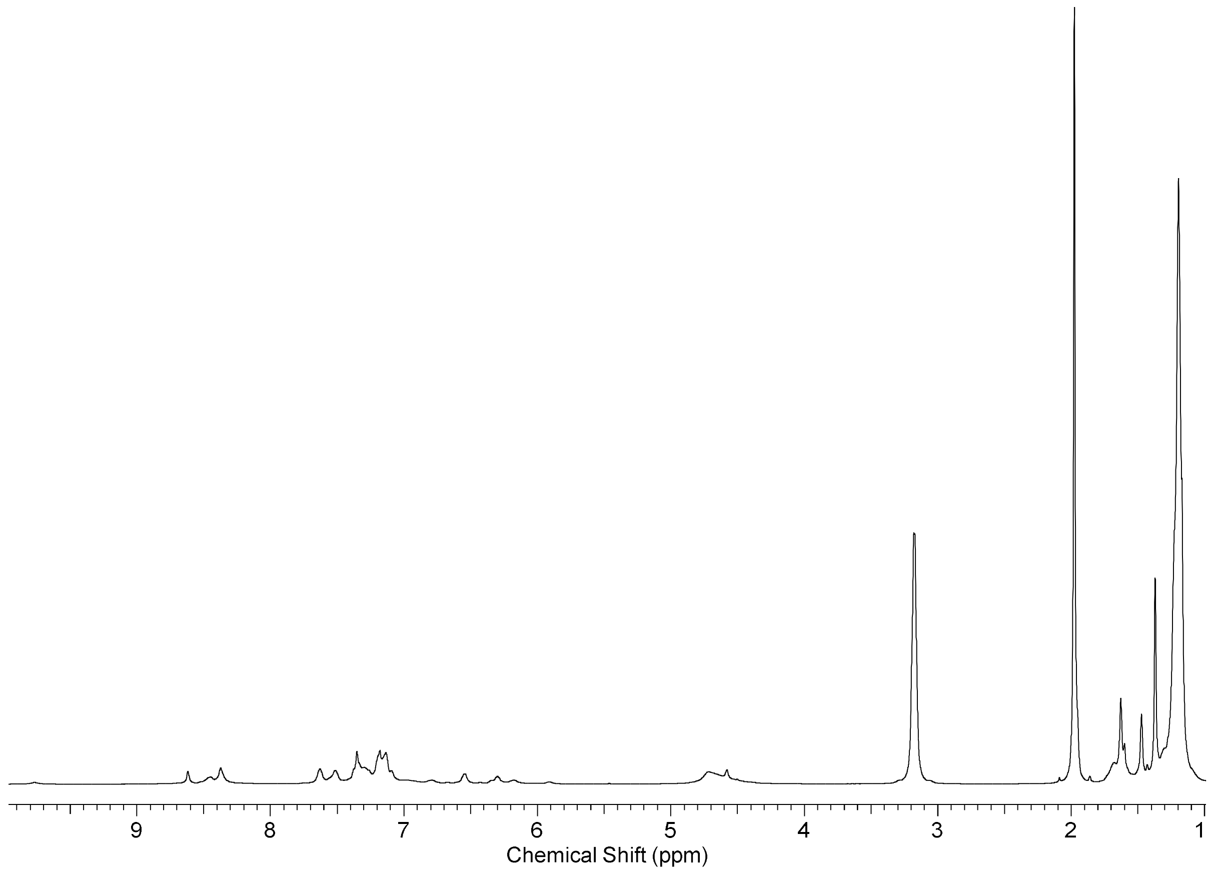

Figure S9. ^{1}H NMR spectrum of the reaction between [Cu(PMe$_3$)(LigH$_2$)](PF$_6$) (**4**(PF$_6$)) with (NEt$_4$)$_2$[MoO$_4$] (CD$_3$CN, 400 MHz).

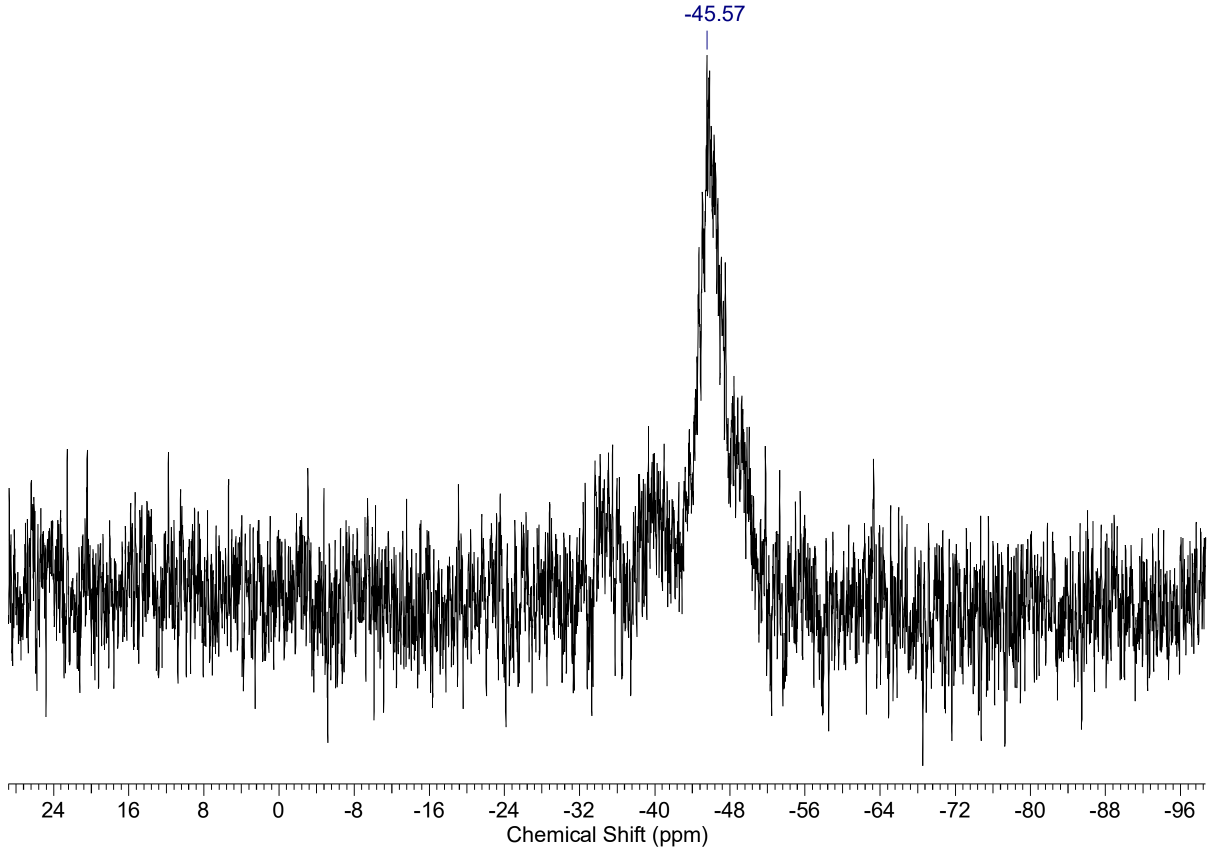

Figure S10. ^{31}P NMR spectrum of the reaction between $[Cu(PMe_3)(LigH_2)](PF_6)$ (**4**(PF_6)) with $(NEt_4)_2[MoO_4]$ (CD_3CN, 400 MHz).

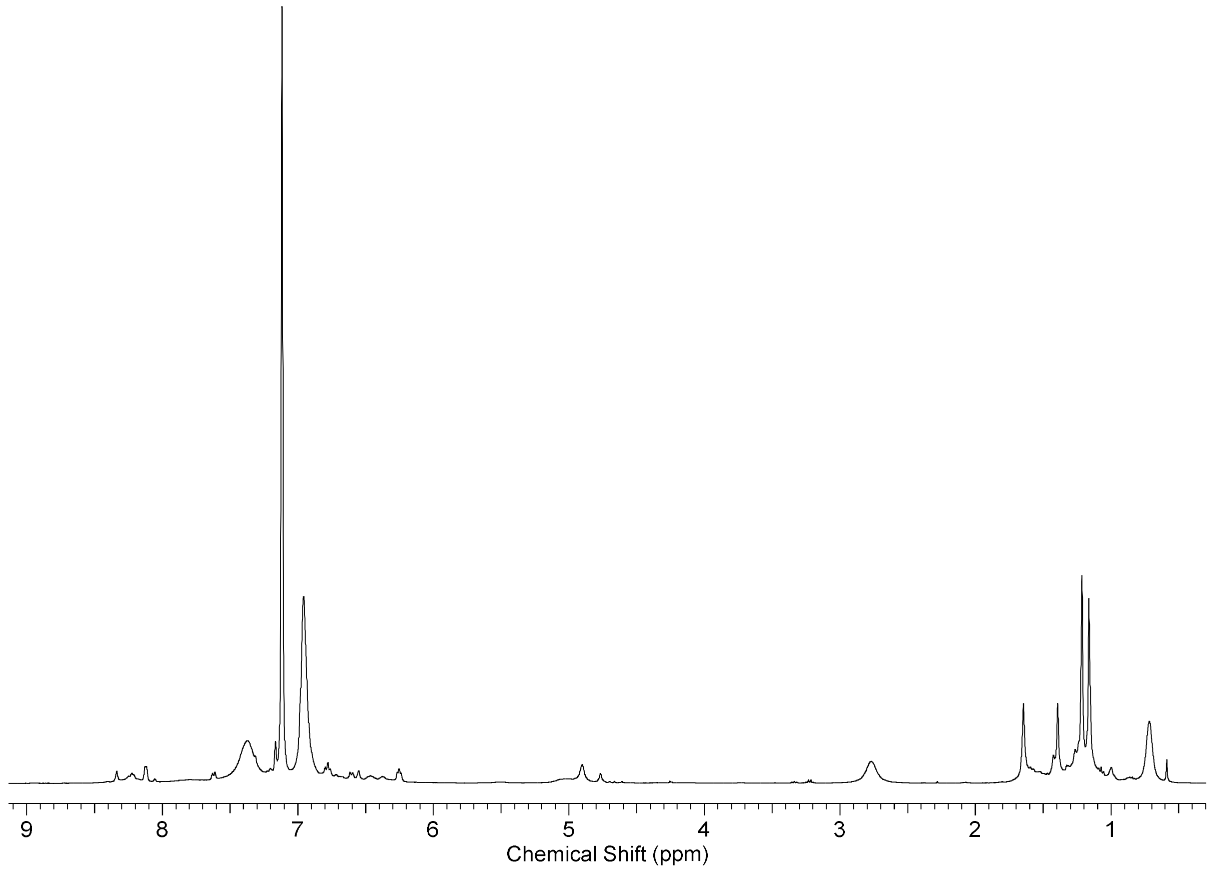

Figure S11. ^{1}H NMR spectrum of the reaction between $[Cu(PPh_3)(LigH_2)](PF_6)$ (**3**(PF_6) and $(NEt_4)_2[MoO_4]$ (CD_3CN, 400 MHz).

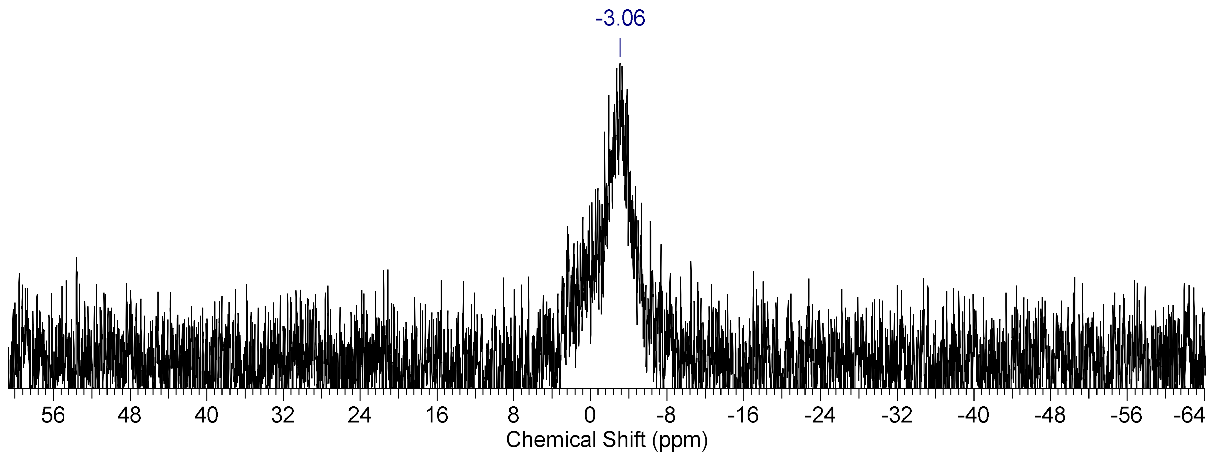

Figure S12. ^{31}P NMR spectrum of the between [Cu(PPh$_3$)(LigH$_2$)](PF$_6$) (**3**(PF$_6$) and (NEt$_4$)$_2$[MoO$_4$] (CD$_3$CN, 400 MHz).

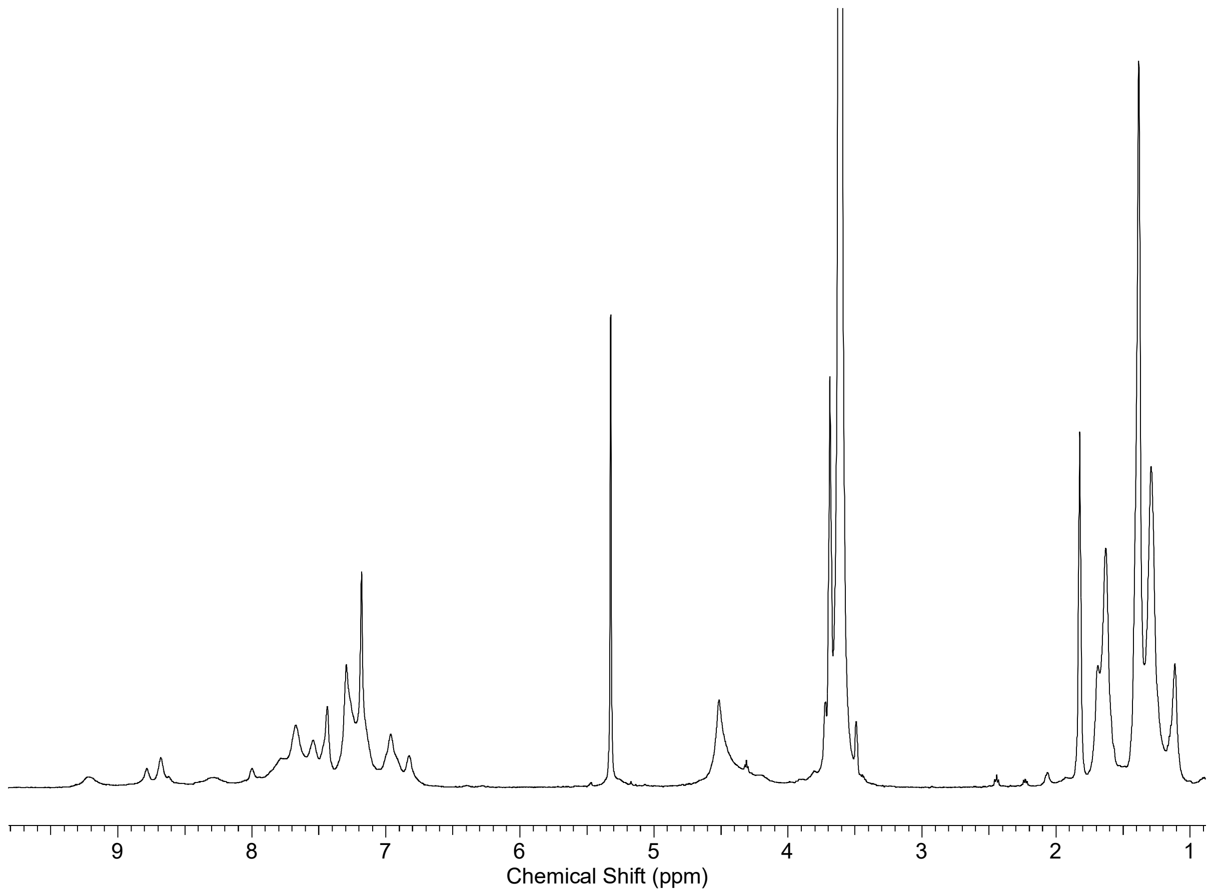

Figure S13. ^{1}H NMR spectrum of the reaction between **1**(PF_6) and NaCN (CD_3CN, 400 MHz).

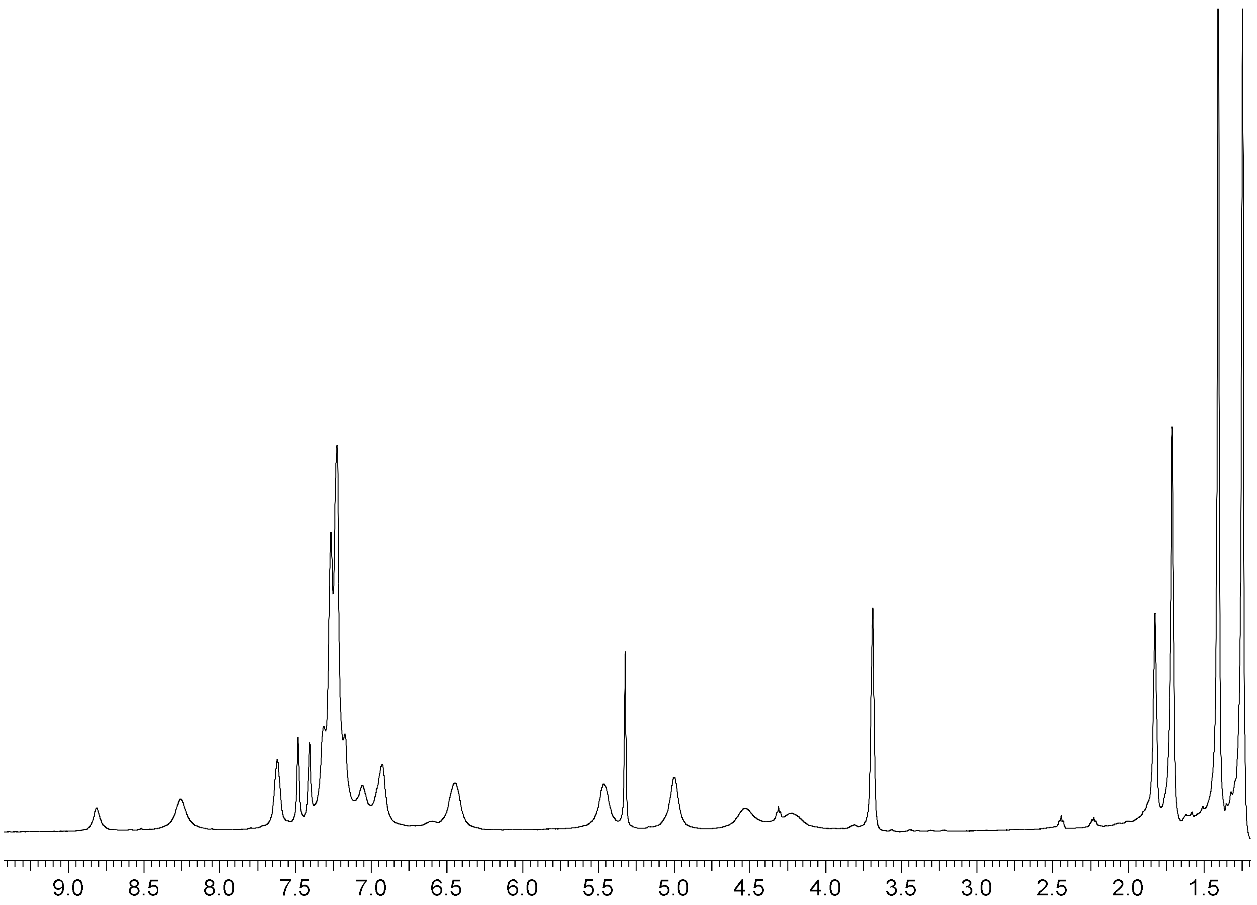

Figure S14. ^{1}H NMR spectrum of the reaction between **1**(PF_6) and styrene (CD_2Cl_2, 400 MHz).

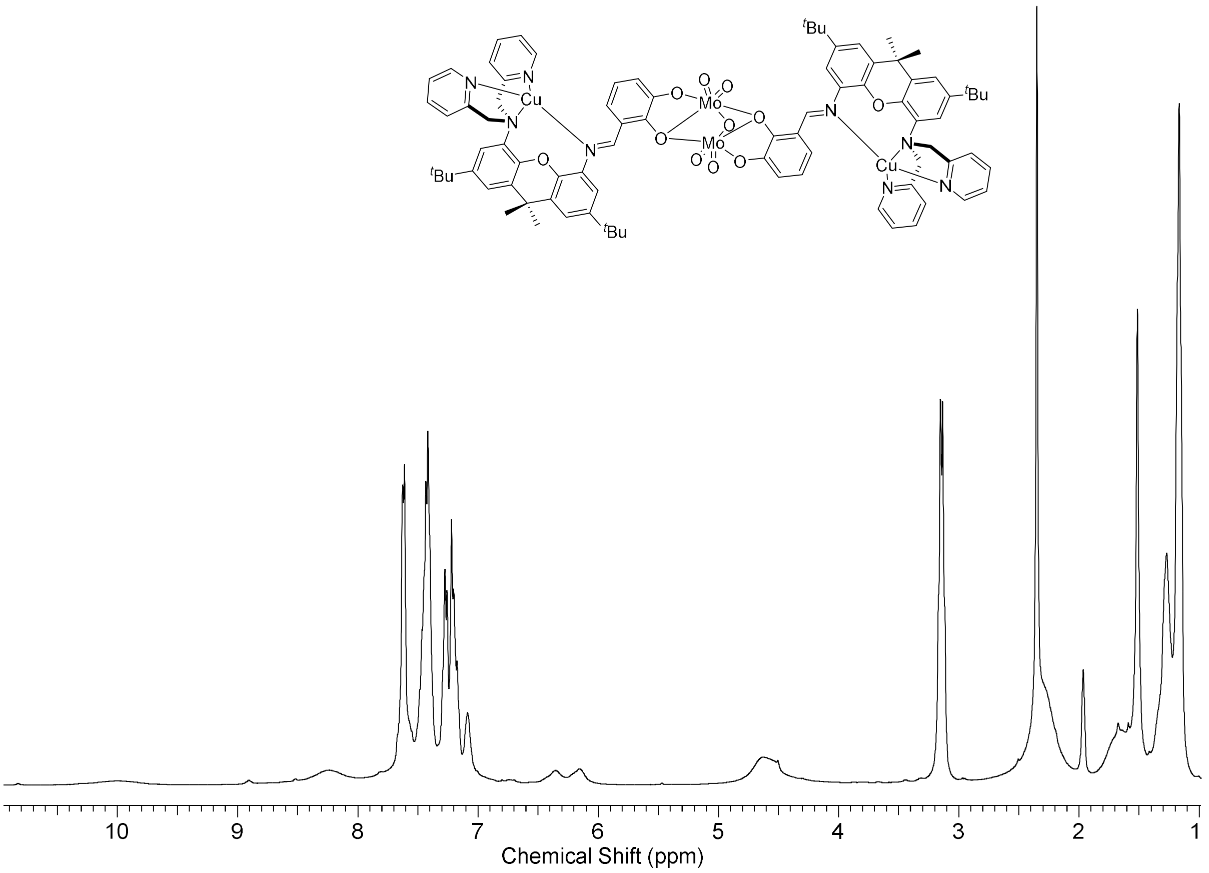

Figure S15. ^{1}H NMR spectrum of $[Cu_2Mo_2O_4(\mu_2\text{-}O)(Lig)_2]$ (**6**) (CD_3CN, 400 MHz).

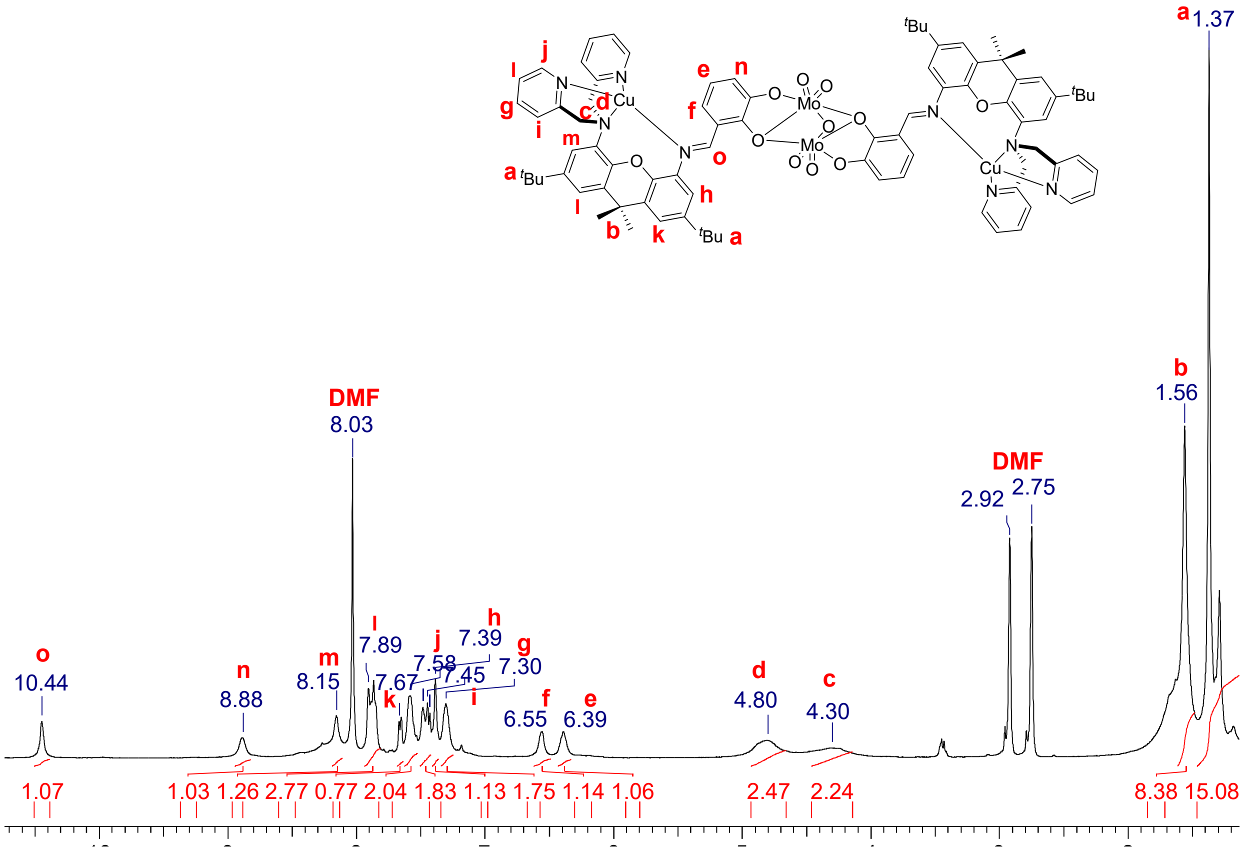

Figure S16. ^{1}H NMR spectrum of $[Cu_2Mo_2O_4(\mu_2\text{-}O)(Lig)_2]$ (**6**) (DMF-d_7, 400 MHz).

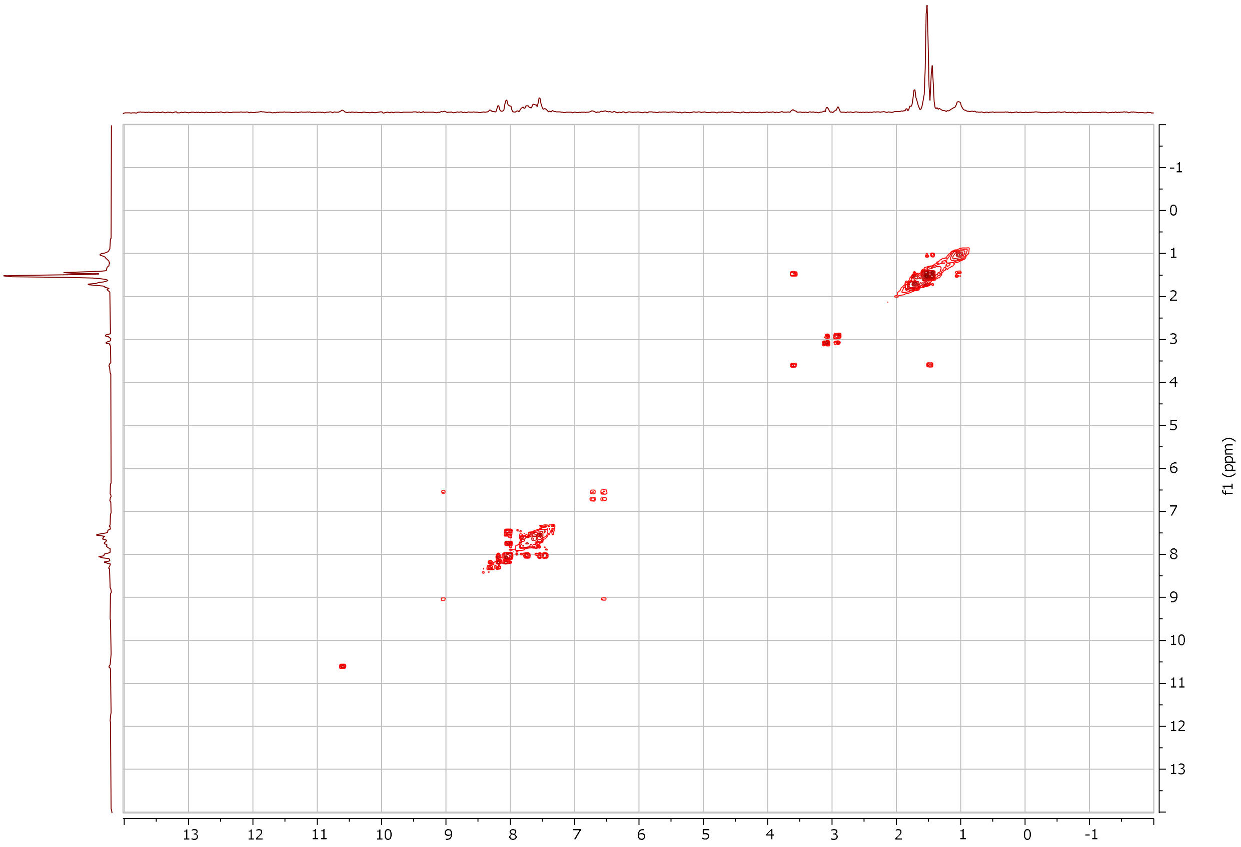

Figure S17. ^{1}H-^{1}H COSY NMR spectrum of $[Cu_2Mo_2O_4(\mu_2\text{-}O)(Lig)_2]$ (**6**) (DMF-d_7, 400 MHz).

2. Mass spectra

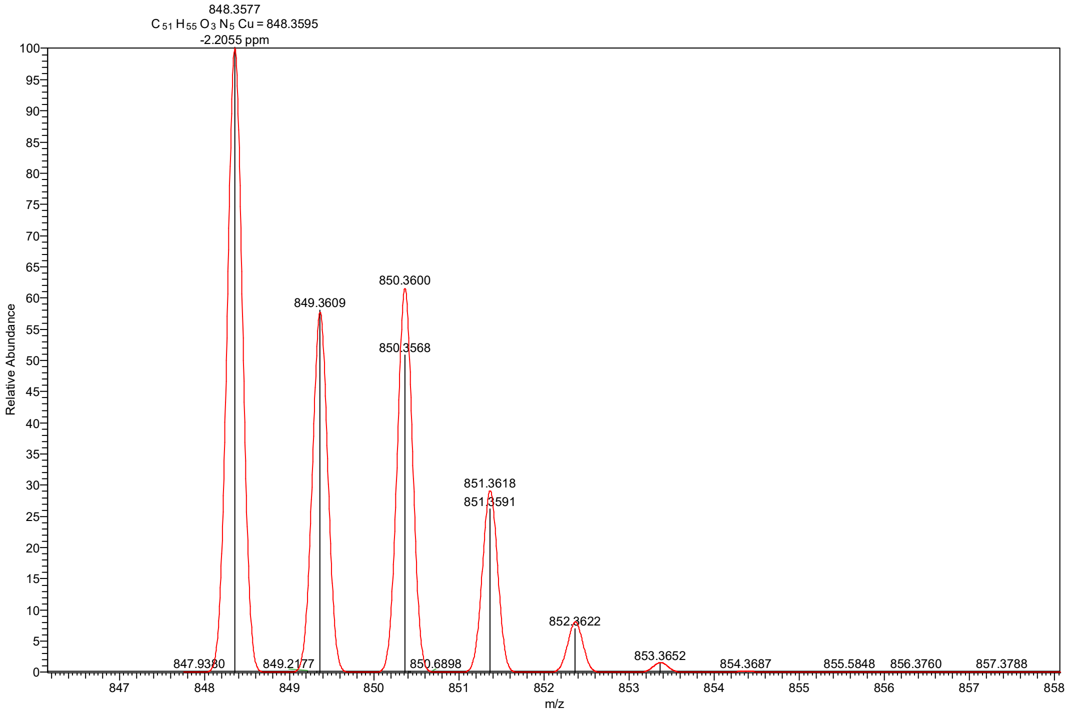

Figure S18. High-resolution mass spectrum of $[Cu(CN(2,6\text{-}Me_2C_6H_3))(LigH_2)]^+$ (**2**$^+$).

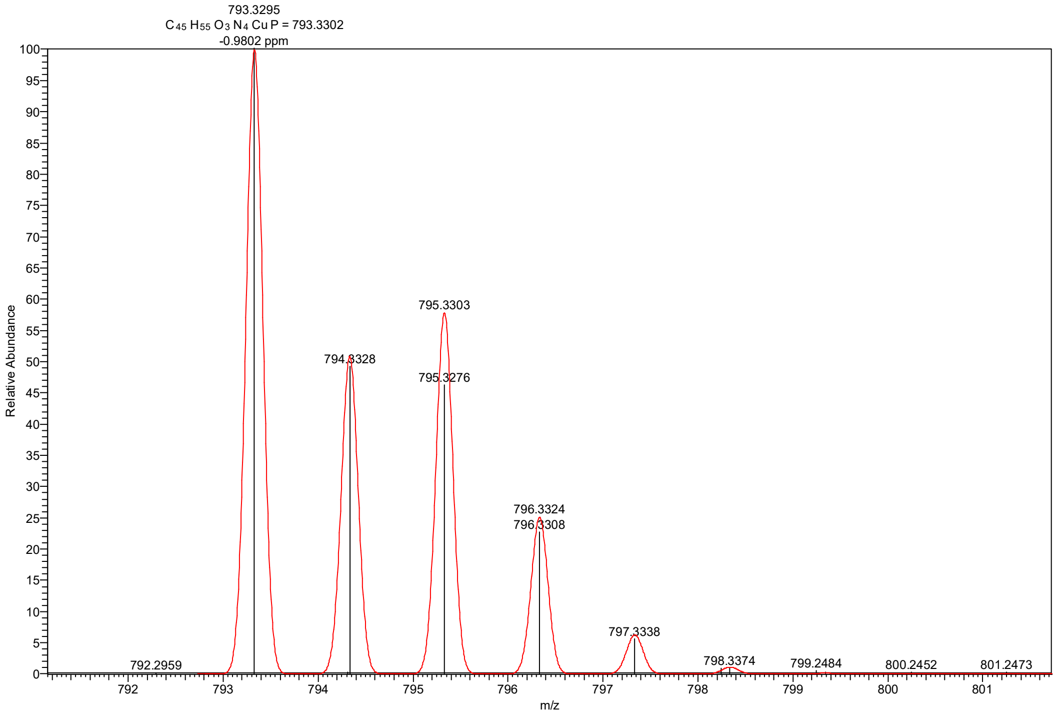

Figure S19. High-resolution mass spectrum of $[Cu(PMe_3)(LigH_2)]^+$ (**3**$^+$).

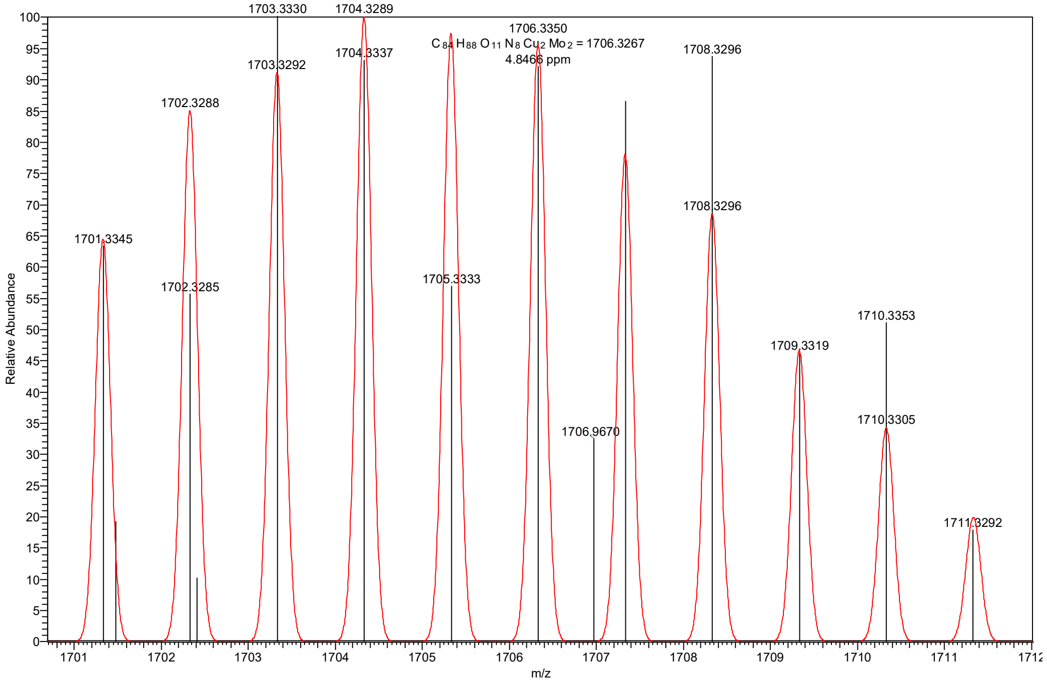

Figure S20. High-resolution mass spectrum of $[Cu_2Mo_2O_4(\mu_2\text{-}O)(Lig)_2]^+$ (**6**$^+$).

3. IR spectra

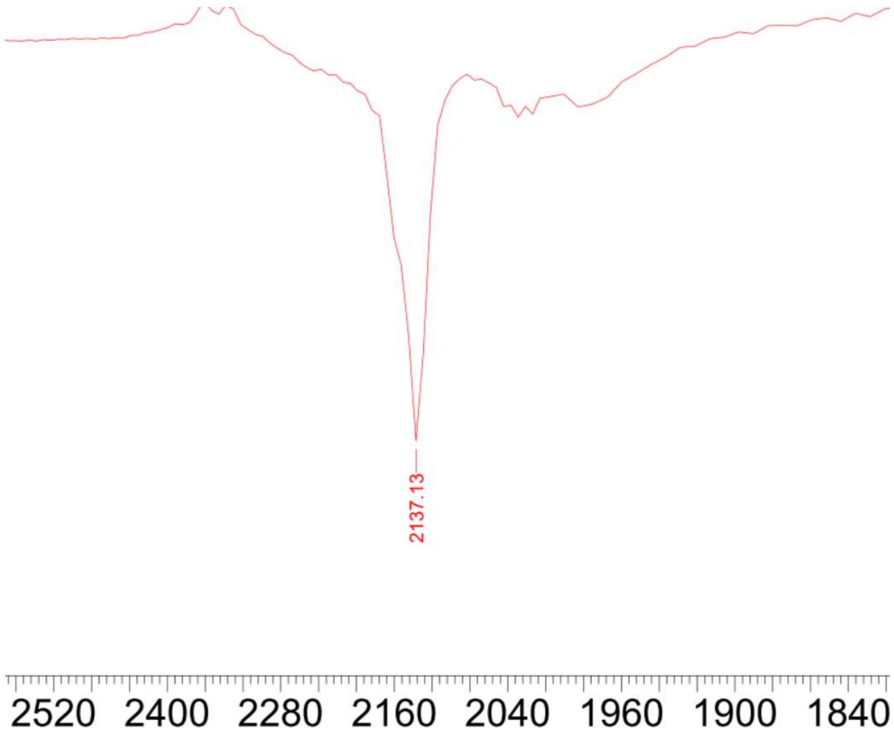

Figure S21. IR spectrum of [Cu(CN(2,6-$Me_2C_6H_3$))(LigH_2)](PF_6) (**2**(PF_6) (isocyanide CN stretch region).

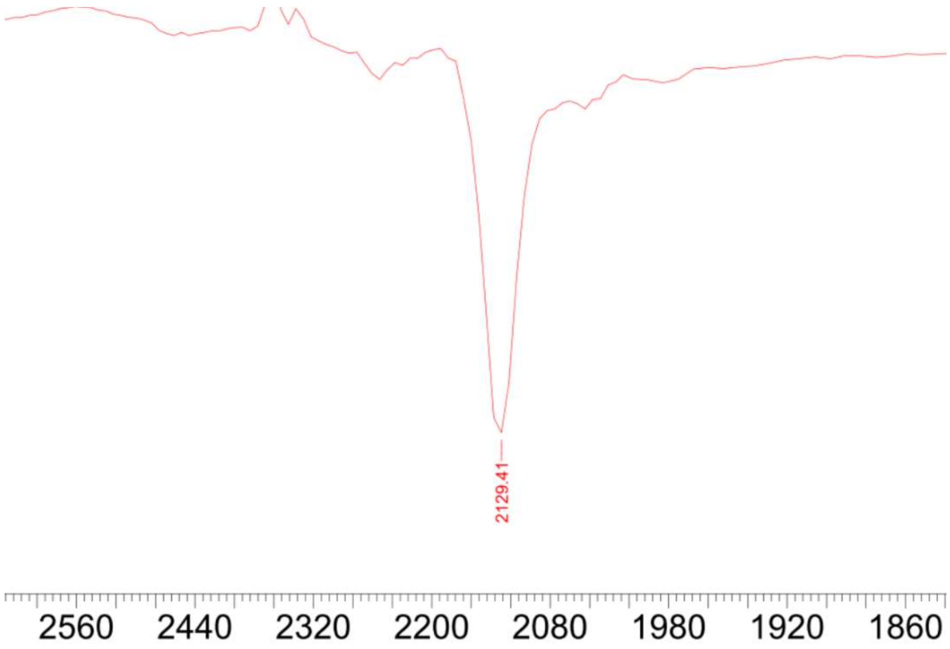

Figure S22. IR spectrum of the reaction product between [Cu(CN(2,6-$Me_2C_6H_3$))($LigH_2$)](PF_6) (**2**(PF_6) and $(NEt_4)_2[MoO_4]$ (isocyanide CN stretch region).
